# Combination of human endothelial colony-forming cells and mesenchymal stromal cells exert neuroprotective effects in the growth-restricted newborn

**DOI:** 10.1038/s41536-021-00185-5

**Published:** 2021-11-18

**Authors:** Kirat K. Chand, Jatin Patel, S. T. Bjorkman, Seen-Ling Sim, Stephanie M. Miller, Elliot Teo, Lara Jones, Jane Sun, Paul B. Colditz, Kiarash Khosrotehrani, Julie A. Wixey

**Affiliations:** 1 UQ Centre for Clinical Research, Faculty of Medicine, The University of Queensland, Brisbane, QLD Australia; 2 The University of Queensland Diamantina Institute, The University of Queensland, Woolloongabba, QLD Australia; 3 Faculty of Health, Queensland University of Technology, School of Biomedical Sciences, Brisbane, QLD Australia; 4 Perinatal Research Centre, Royal Brisbane and Women’s Hospital, Brisbane, QLD Australia

**Keywords:** Stem cells, Development of the nervous system

## Abstract

The foetal brain is particularly vulnerable to the detrimental effects of foetal growth restriction (FGR) with subsequent abnormal neurodevelopment being common. There are no current treatments to protect the FGR newborn from lifelong neurological disorders. This study examines whether pure foetal mesenchymal stromal cells (MSC) and endothelial colony-forming cells (ECFC) from the human term placenta are neuroprotective through modulating neuroinflammation and supporting the brain vasculature. We determined that one dose of combined MSC-ECFCs (cECFC; 10^6^ ECFC 10^6^ MSC) on the first day of life to the newborn FGR piglet improved damaged vasculature, restored the neurovascular unit, reduced brain inflammation and improved adverse neuronal and white matter changes present in the FGR newborn piglet brain. These findings could not be reproduced using MSCs alone. These results demonstrate cECFC treatment exerts beneficial effects on multiple cellular components in the FGR brain and may act as a neuroprotectant.

## Introduction

Foetal growth restriction (FGR) occurs in around 3–15% of pregnancies with even greater rates in developing countries^1–3^. FGR is often caused by placental insufficiency, resulting in an inadequate supply of oxygen and nutrients to the developing foetus in utero^4^. The foetal brain is particularly vulnerable to FGR conditions and subsequent abnormal neurodevelopment is common^5^. Adverse neurodevelopmental outcomes include learning and behavioural disorders and cerebral palsy, which have lifelong medical and financial consequences^6–10^. Due to medical advances, more FGR babies now survive, although many remain at risk of these neurodevelopmental disabilities. No treatments currently exist to protect the FGR newborn brain.

Both neuronal and white matter alterations are observed in both the human FGR infant and animal models of FGR^11–16^. Recent animal studies have shown that inflammation plays a key role in these alterations^17–19^. High concentrations of pro-inflammatory cytokines have been reported in the blood of preterm FGR infants two weeks after birth^20^. Increases in pro-inflammatory cytokines are correlated with adverse neurological outcomes at 2 years of age in preterm small for gestational age newborns^21^. An increase in inflammatory mediators in the blood may affect the brain because of the disruption to the structural and functional integrity of the neurovascular unit (NVU) in the FGR newborn^22–24^ and toxic mediators may pass freely in and out of the brain. Therapeutic targeting of inflammatory pathways and the NVU hold promise as neuroprotectants in FGR newborns^25^.

The NVU separates the brain from the blood circulation and is composed of vascular endothelial cells, glial cells (astrocytes and microglia), neurons, pericytes and the basement membrane. The cells of the NVU share close and complex interactions that are crucial in maintaining blood–brain barrier (BBB) integrity and cerebral homoeostasis, ensuring healthy brain development. Disruption to the NVU plays an essential role in progression of numerous central nervous system (CNS) pathologies allowing toxic substances, such as pro-inflammatory cytokines to infiltrate the brain, which can exacerbate neuroinflammation and injure neurons and white matter^26,27^. As the NVU is developed in the newborn, maintaining the structural and functional integrity of the NVU is likely an essential element in neuroprotection in the newborn.

Endothelial colony forming cells (ECFCs) are the vascular precursors, devoid of hematopoietic and myeloid contamination^28^. They reside throughout the vasculature, have self-renewal as well as a capacity to engraft forming both de novo neo-vessels as well as chimeric vessels within ischaemic sites of the host, facilitating reperfusion and initiating tissue regeneration^29,30^. They have been isolated from numerous sources, classically the umbilical cord blood (UCB) and the term placenta^31,32^. The vasculogenic capacity of ECFCs is greatly enhanced upon co-incubation or co-delivery with mesenchymal stromal cells (MSCs), a process known as priming^33^. Moreover, engraftment of ECFCs if combined with MSCs (cECFC) can bypass the host immune system^34^. Recent evidence suggests that cECFC outperformed MSCs alone in reperfusing ischaemic limbs through neo-vascularisation^29^. Additionally, this combination of cECFCs did not require additional immunosuppressive therapy in immunocompetent animals^29^ highlighting their potential use as an allogeneic off-the-shelf cell therapy.

In the present study, we obtained pure foetal MSC and ECFC from the healthy human term placenta^32,35^. We hypothesised that the cECFC treatment provides neuroprotection in the FGR newborn. Using our established preclinical piglet model of FGR^17,36,37^, we assessed whether one dose on the first day of life in a term FGR piglet would regenerate damaged vasculature, restore the NVU, reduce brain inflammation and improve adverse neuronal and white matter changes present in the FGR newborn piglet brain.

## Results

### Physiological parameters of FGR and normally grown piglets

Body weight and brain weight were significantly lower for all FGR piglet groups at postnatal day 4 (P4) compared with normally grown (NG) piglets (body *p* < 0.0001; brain *p* < 0.05; Table 1). Piglets in all FGR groups were asymmetric indicated by a mean brain to liver weight ratio (B:L) > 1. There was no significant difference in body weight (*p* = 0.735), brain weight (*p* = 0.999) or liver weight (*p* = 0.721) between NG piglet groups (Table 1).

**Table 1:** Physiological parameters in FGR and NG piglets.

	NG (*n* = 8)	FGR (*n* = 8)	FGR + cECFC (*n* = 8)	NG + cECFC (*n* = 7)	FGR + MSC (*n* = 5)
Body weight in grams (mean ± SEM)	1968 ± 138.10	1023 ± 77.13****	1028 ± 88.96****	1743 ± 91.59	800 ± 35.78****
Brain weight in grams (mean ± SEM)	32.74 ± 0.50	29.97 ± 0.60*	29.38 ± 1.18*	32.03 ± 0.59	28.87 ± 0.82*
Liver weight in grams (mean ± SEM)	51.24 ± 6.16	24.19 ± 1.86****	25.22 ± 2.77****	42.66 ± 2.94	21.49 ± 1.73****
Brain:liver ratio (mean ± SEM)	0.69 ± 0.07	1.30 ± 0.12**	1.29 ± 0.17**	0.78 ± 0.06	1.38 ± 0.11**
Mortality	0/8	1/9	0/8	0/7	0/5

Piglet body weight, brain weight, liver weight, temperature, and mortality. Mean body weight was significantly lower in all FGR groups compared with NG piglets. Brain weight was significantly reduced in all FGR groups compared with the NG group. Mean brain to liver weight ratio was significantly higher in all FGR groups compared with NG piglets indicating asymmetric growth restriction in the FGR piglets. Values are the mean ± SEM. *p < 0.05; **p < 0.01; ****p < 0.0001 versus NG.

### cECFC delivery and distribution

Placental ECFC and MSC were obtained from three donors. 20 piglets received intravenous (i.v.) stem cells (either cECFC or MSC) versus Sham. Core body temperature was regularly monitored following stem cell administration, with altered body temperature being a hallmark of immunological or inflammatory response. No differences in body temperature (°C) were evident between any of the five experimental groups (NG 38.33 ± 0.14; FGR 38.39 ± 0.08; FGR + cECFC 38.15 ± 0.08; NG + cECFC 38.2 ± 0.09; FGR + MSC 38.03 ± 0.21; average over 3 days). Normal temperature for a piglet is approximately 38.5 °C^38^. Piglets in all five groups responded well to feeds with one death (due to inability to thrive) in the FGR group (Table 1).

**Fig. 1:**
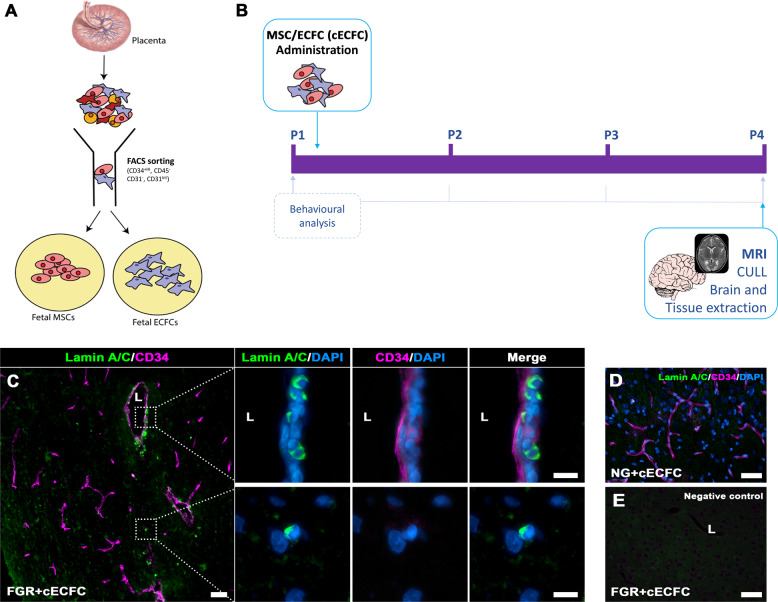
Isolation and administration of combined Mesenchymal and Endothelial Colony Forming Cells. **A** Foetal MSC and ECFCs were derived and isolated from healthy human placenta via FACs sorting. Isolated cells were cultured, enriched and prepared for intravenous administration as a combined stem cell preparation, termed cECFC (10^6^ MSC and 10^6^ ECFC). **B** Schematic representation of cECFC therapy in the newborn FGR pig model. **C** Presence of cells in treated FGR brain was confirmed post-mortem (P4) using Lamin A/C. Lamin A/C-positive cells were observed in the parenchyma as well as instances of vessel engraftment (lumen; L). **D** No Lamin A/C-positive labelling was observed in NG + cECFC tissue and **E** negative control (primary antibody omitted) (Scale bars: 50 µm; **C** high magnification: 10 µm).

Biodistribution of human stem cells was detected in the piglet brain at P4 based on labelling with a human-specific Lamin A/C antibody. Cells positive for Lamin A/C were found to engraft into the vasculature of microvessels as well as sporadic instances of parenchymal labelling in FGR + cECFC brain (Fig. 1C). No Lamin A/C labelling was observed in the technical control (no primary antibody) nor in the NG + cECFC piglet brain (Fig. 1D, E). Overall, cECFC delivery was deemed safe and distributed to the vasculature and perivascular areas of the FGR brain.

### cECFC administration promotes vessel density

**Fig. 2:**
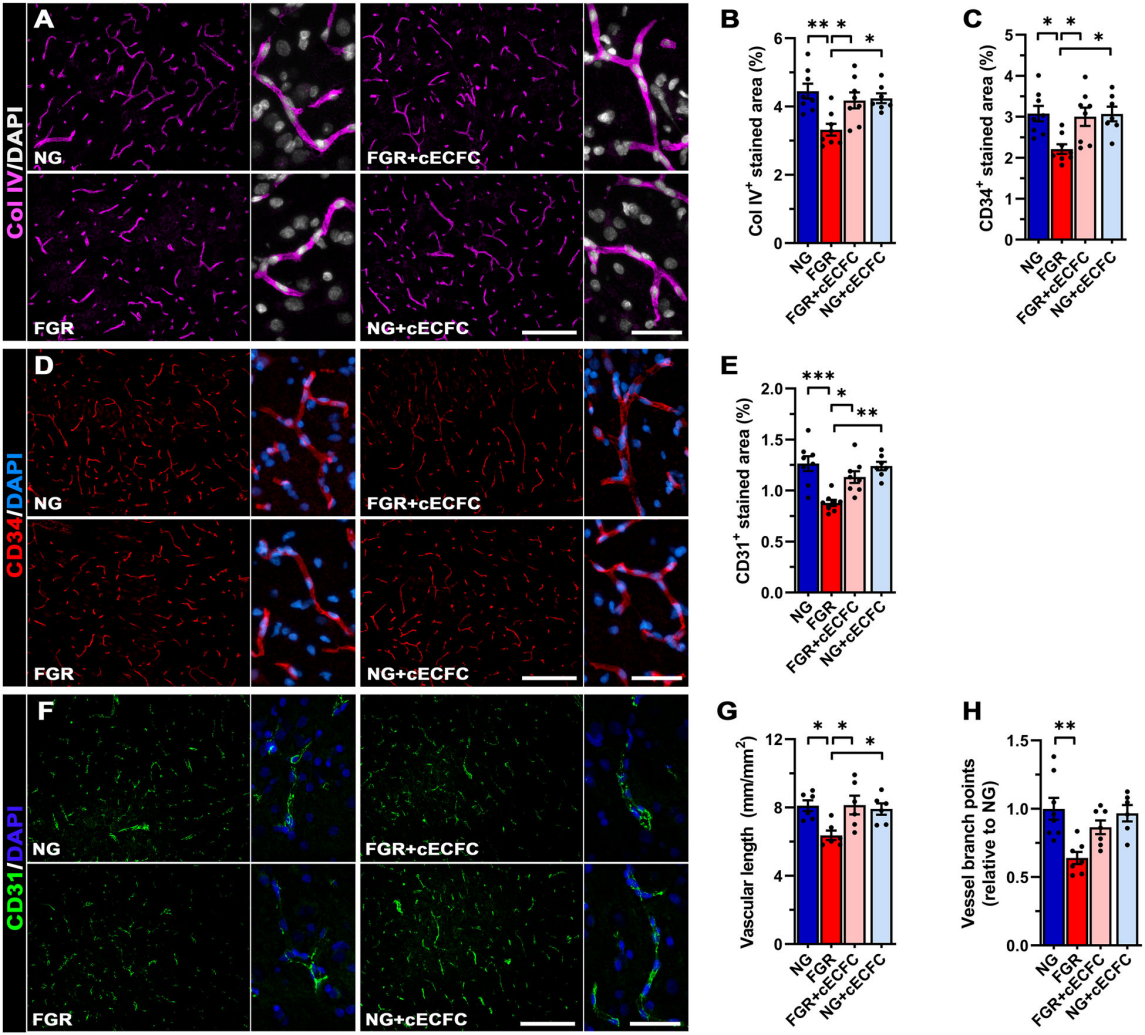
cECFC administration promotes vessel density and reduces BBB-permeability. **A** Representative immunofluorescent labelling of vessel basement membrane (Col IV; magenta) in the parietal cortex. FGR displayed truncated vasculature with limited branching compared with other groups. **B** Analysis found a significant decrease in coverage of Col IV-positive vessels which improved following cECFC administration. **D** Vascular endothelial progenitor cell marker (CD34; red) showed similar patterns observed as Col IV, with FGR demonstrating lower labelled area (**C**). FGR displayed decreased labelling of the endothelial cell marker CD31, which was higher in cECFC treated brains (**E** and **F**). **G** Analysis of the vasculature showed a significant decrease in vessel length as assessed by labelling of Col IV. **H** Vessel branching was also reduced in FGR relative to NG. FGR + cECFC did not display increased vascular branching. All values are expressed as mean ± SEM (minimum *n* = 6 for all groups). Two-way *ANOVA* with Tukey post-hoc test (*p < 0.05, **p < 0.01) (Scale bars: low magnification: 200 µm; high magnification: 50 µm).

We investigated the potential benefits of cECFCs in improving vasculature impairment of the FGR brain. A key component of the cerebrovasculature is collagen IV (Col IV), which contributes to ~50% of the basement membrane of the NVU. All groups displayed robust labelling of Col IV across the length of the vasculature, however analysis of area labelled showed a significant reduction in the FGR brain compared with NG (3.32 ± 0.17% *c.f*. 4.45 ± 0.22%, *p* = 0.009; Fig. 2A, B) indicating a reduction in vascularisation. cECFC-treated FGR brain displayed higher vessel density (4.18 ± 0.24%) compared with FGR (*p* = 0.048; Fig. 2A, B). Administration of MSC had no significant effect on vessel density in FGR (Supp. Fig. 1A, B). Analysis of the CD34, a marker of hematopoietic stem cells and vascular endothelial progenitor cells^39^, closely reflected the observations of Col IV labelling with significantly less labelling in FGR brains (2.21 ± 0.12%) compared with NG (3.08 ± 0.19%) (*p* = 0.019; Fig. 2C, D), that was normalised with cECFC treatment (3.00 ± 0.22%). The endothelial cell marker CD31 was decreased in FGR compared with NG (0.88 ± 0.03% c.f. 1.26 ± 0.07%, *p* = 0.0003), and significantly recovered in FGR following cECFC treatment (*p* = 0.0165; Fig. 2E, F). Examination of vascular length and vascular complexity based on the number of branch points from the primary vessel revealed a significant decrease in the total vascular length in FGR brain compared with NG (*p* = 0.025) with significant restoration upon cECFC treatment (*p* = 0.021; Fig. 2G). In addition to loss in vessel length we also observed a decrease in vessel branch points in FGR brain relative to NG (*p* = 0.002; Fig. 2H). cECFC treatment partially improved vessel branching in FGR + cECFC brains appearing comparable to that observed in NG brain (FGR + cECFC *c.f*. NG, *p* = 0.405). Although we could not find any evidence of cells in the NG + cECFC piglet brain comparison of NG and NG + cECFC of vessel length and branching showed no differences suggesting cECFC administration does not promote unwarranted angiogenic effects (Fig. 2). These findings support the potential of cECFC as a safe treatment to improve cerebrovascularisation in the FGR neonate as previously shown in other ischaemic scenarios.

### cECFC treatment enhances neurovascular unit integrity

**Fig. 3:**
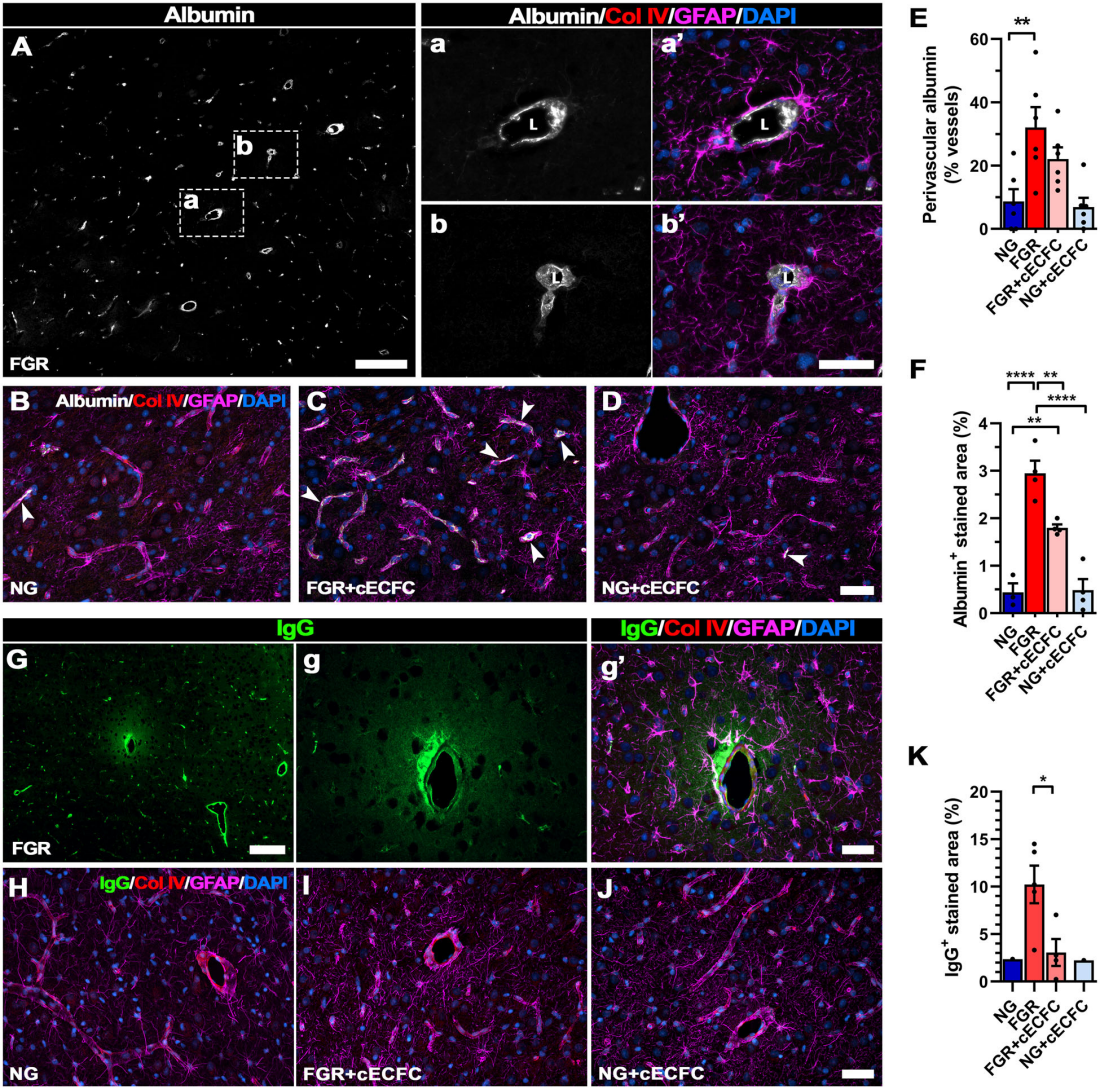
cECFC administration ameliorates neurovascular integrity in the FGR neonate. Representative labelling of endogenous albumin in the FGR as a marker of altered blood brain barrier integrity. **A** FGR brain showed albumin labelling (grey) predominantly localised to the perivascular space, between the lumen (L) and astrocyte endfeet (GFAP; magenta) (see **Aa’-Ab’**). **B** NG, **C** FGR + cECFC and **D** NG + cECFC displayed lower less overt albumin labelling. **E** A significantly higher percentage of vessels in FGR brain displayed perivascular labelling compared with NG. cECFC treatment did not significantly reduce the number of vessels with perivascular labelling. **F** FGR brain presented significantly greater albumin-positive labelling compared with NG. FGR + cECFC showed less labelling but was still elevated compared with NG. **G** Representative labelling of IgG (green) in the FGR brain showed extravasation into the brain parenchyma. Evident astrocyte activation (GFAP; magenta) was observed at vessels displaying altered permeability. **H** NG and **J** NG + cECFC displayed minimal IgG extravasation and maintained strong astrocyte interaction at the cerebrovasculature. **I** FGR + cECFC demonstrated less frequent IgG extravasation and significantly lower IgG-positive labelled area compared with untreated FGR **K** All values are expressed as mean ± SEM (minimum *n* = 6 for all groups). For **K** only brains pigs demonstrating IgG extravasation were included, NG (*n* = 1), FGR (*n* = 7), FGR + cECFC (*n* = 6), NG + cECFC (*n* = 1). Two-way *ANOVA* with Tukey post-hoc test (**p < 0.01, ****p < 0.001). For **K**; unpaired Student’s *t* test (*p < 0.05) (Scale bars: 50 µm; for **A** and **G**: low magnification: 200 µm).

We further investigated whether the improved vascular structure and organisation in the FGR brain that occurred with cECFC treatment, resulted in maintenance of BBB integrity. Previous studies have demonstrated the utility of endogenous serum proteins as markers of altered BBB-permeability. We used albumin (~69 kDa) and IgG (~155 kDa) extravasation to assess whether cECFC-treated animals demonstrated improvement to BBB permeability. FGR brains displayed predominantly perivascular labelling of albumin (Fig. 3A) with no observed extravasation into the parenchyma. Astrocyte end-feet encasing vessels with perivascular albumin presented activated morphology, with hypertrophic end-feet and swelling of cell bodies (Fig. 3Aa, b’). In comparison, NG and cECFC-treated groups displayed low intensity albumin labelling that was predominantly restricted to the vessel lumen and a return of juxtavascular astrocytes to a ramified astrocyte morphology (Fig. 3B, C, D). Quantification of albumin labelling showed a greater number of vessels with perivascular labelling in FGR (32.04 ± 6.45%) compared with NG (8.70 ± 3.84%) (*p* = 0.007; Fig. 3E). Albumin-positive labelled area, used to assess the aggregation of albumin in the perivascular space, was significantly higher in FGR when compared with NG (2.90 ± 0.26% c.f. 0.44 ± 0.19% respectively, *p* < 0.0001) and was significantly reduced upon cECFC treatment (*p* = 0.009; Fig. 3F). However despite cECFC treatment, albumin in the perivascular space remained significantly greater when compared with NG (1.80 ± 0.07 % c.f. 0.44 ± 0.19% respectively, *p* = 0.005; Fig. 3F).

Similarly, we examined permeability of the BBB with the endogenous immunogobulin IgG which is a larger molecular weight protein. Labelling of IgG showed sporadic extravasation into the parenchyma of FGR brains (5/7 examined, with 28/56 fields displaying extravasation), with juxtavascular astrocytes in these regions displaying activated morphology and reduced end-feet contact with the vessel (Fig. 3G-g’). NG and NG + cECFC groups showed limited to no IgG labelling and were therefore excluded from statistical analyses (1 animal displayed extravasation in both NG & NG + cECFC; Fig. 3H, J). FGR + cECFC brains showed fewer incidences of extravasation (4/8 brains examined, with10/64 fields displaying extravasation), and significantly decreased IgG-labelled area (Fig. 3I, K).

**Fig. 4:**
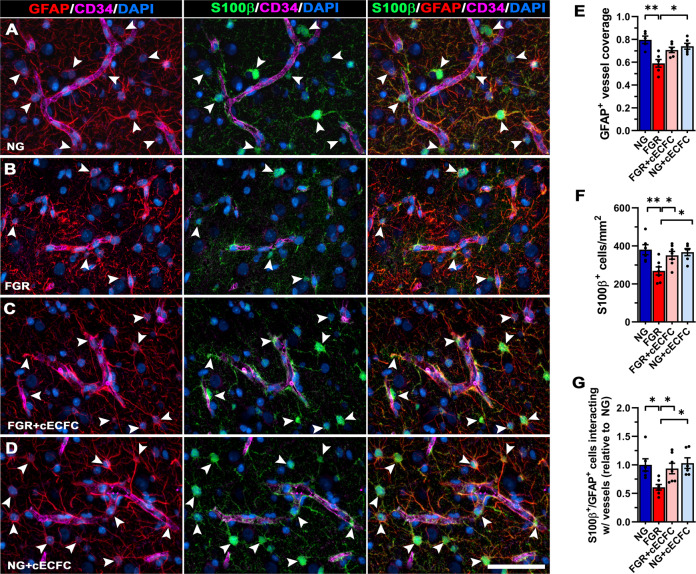
Recovery of mature astrocytes at the NVU of FGR brain following cECFC administration. Representative labelling for pan-astrocyte marker (GFAP; red) and mature astrocyte marker (S100β; green) in the cortex of pig at day 4. **A** NG showed strong S100β labelling co-localised to GFAP (arrowheads). These mature astrocytes demonstrate strong interaction of end-feet enveloping the length of neurovasculature. **B** FGR displayed intense thickened process labelling around the vasculature, indicative of reactive state. Less frequent S100β labelling was observed in the cortex as well as at the vasculature specifically. GFAP labelling along the vasculature was more sporadic and uneven when compared with NG. **C** FGR + cECFC displayed mature astrocytic labelling comparable to NG, with astrocyte end-feet displaying more contact with vasculature. **D** NG cECFC treated showed no alteration in cell count or morphology. **E** Co-localisation analysis demonstrated a decrease in GFAP-positive vessel coverage in FGR brain compared with NG. **F** The reduction in S100β cell counts in FGR was ameliorated following cECFC treatment. **G** Quantification of S100β-GFAP positive cells interacting with the vasculature confirmed a significant decrease in FGR relative to NG. FGR + cECFC displayed similar numbers of S100β-GFAP positive cells interacting with the vasculature to that observed in NG. All values are expressed as mean ± SEM (minimum *n* = 5 for all groups). Two-way *ANOVA* with Tukey post-hoc test (*p < 0.05) (Scale bars: 50 µm).

Reduction in vessel-astrocyte interaction may be associated with less mature astrocytes at the vessel–glia interface in the FGR newborn brain. We therefore co-labelled for S100β (marker of mature astrocytes) and GFAP to investigate altered astrocyte interaction with microvessels. In NG animals, we observed an abundance of S100β-positive juxtavascular astrocytes and co-localisation with GFAP with consistent contact of end-feet with the vasculature (Fig. 4A). FGR demonstrated altered labelling patterns, with significantly less total S100β^+^ cells and reduced S100β-GFAP positive cells along the vasculature compared with NG animals (*p* = 0.007; Fig. 4B, F, G). This corresponded to an overall decrease in GFAP-positive vessel coverage (*p* = 0.004; Fig. 4E). FGR + cECFC displayed labelling comparable to that observed in NG, with an abundance of S100β-GFAP-positive labelling at the vasculature (Fig. 4C). FGR + cECFC showed similar GFAP coverage to that of NG brains, with a strong trend towards improving astrocyte coverage when compared with FGR (*p* = 0.056; Fig. 4E). These findings suggest that cECFC administration on first day of life promotes the maturation of juxtavascular astrocytes.

### cECFC treatment attenuates glial cell activation and modifies the pro-inflammatory environment of the FGR brain

**Fig. 5:**
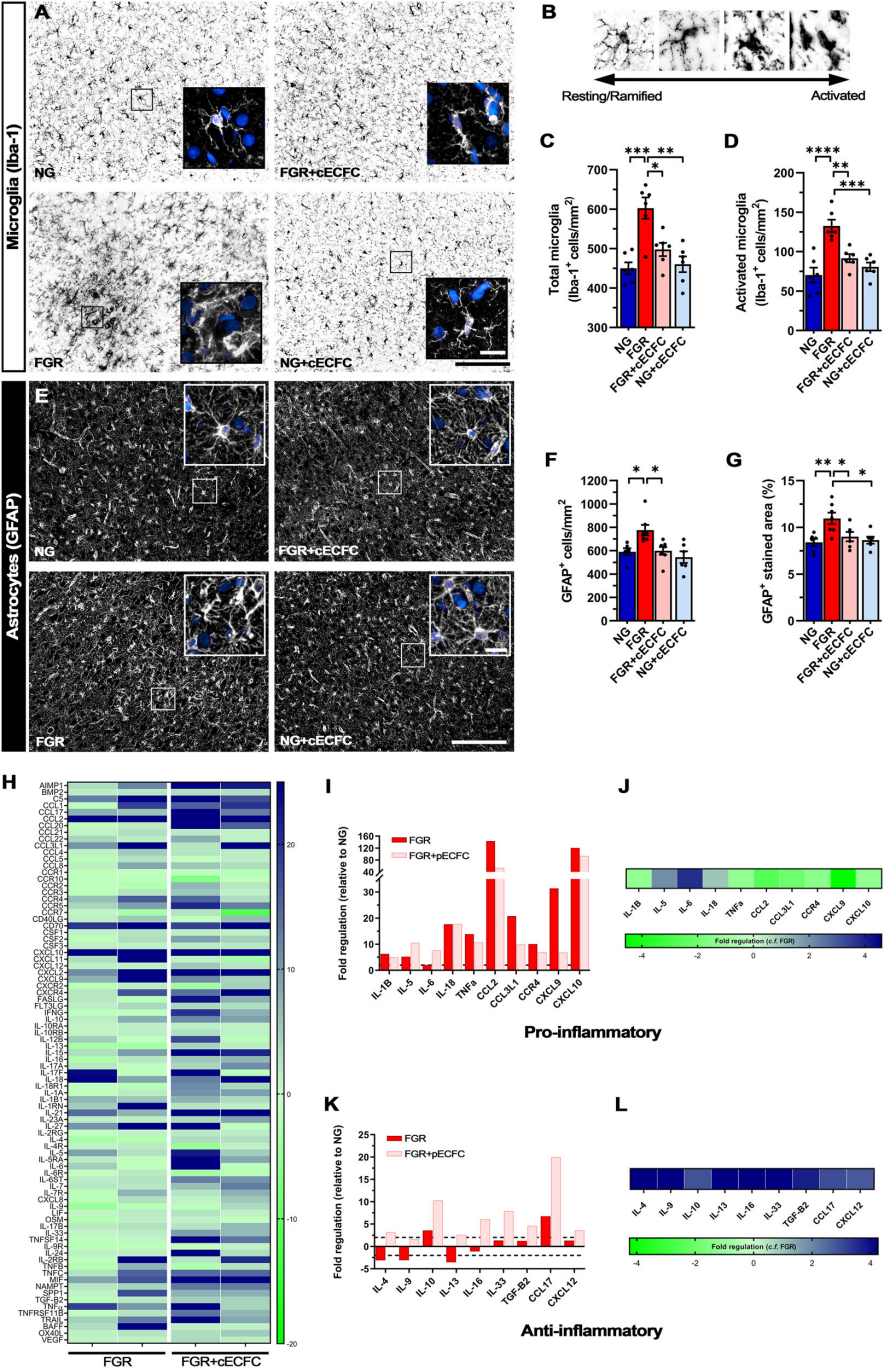
cECFC treatment attenuates glial activation in FGR brains. Representative labelling of microglial cells (Iba-1; black) in the parietal cortex. **A** NG brains display typical ramified microglial morphology, with even distribution, small cell bodies, and extended processes (see insert). FGR demonstrate significant activation of microglia with dense cellular bodies, retraction and thickening of processes, and a high degree of cellular overlap. cECFC-treated animals displayed morphology comparable to NG. **B** Microglia were assigned as resting or ramified based on well-established morphology associated with activation state of glial cells. **C** FGR demonstrated significantly higher total number of Iba-1 positive cells and activated microglia (**D**). Similar trends were observed in the white matter of FGR brain (see Supp. Fig. 3). **E** Astrocytes (GFAP; white) showed evident alterations in morphology. NG displayed typical star like cells with long process extensions. FGR displayed activated astrocyte morphology, with retracted processes and enlargement of cell bodies. cECFC-treated animals displayed similar morphology to NG. **F** cECFC treatment reduced the number of astrocytes in the cortex toward levels comparable to other groups. **G** Treatment with cECFCs reduced astrocyte activation as assessed with GFAP-positive stained area. **H** Heat map of porcine inflammatory cytokine and receptors arrays demonstrates altered expression of pro- and anti-inflammatory mediators in the cortex of FGR relative to NG. FGR + cECFC showed alterations in inflammatory genes relative to NG and untreated FGR (*n* = 8 for all groups, pooled samples of *n* = 4 for each group per array). Expression of well-characterised pro-inflammatory **I** and anti-inflammatory **K** genes relative to NG. FGR + cECFC displayed down-regulation of pro-inflammatory (**J**) and up-regulation of key anti-inflammatory genes (**L**) when compared with untreated FGR. All values are expressed as mean ± SEM (minimum *n* = 6 for all groups). Two-way ANOVA with Tukey post-hoc test (*p < 0.05, **p < 0.01, ***p < 0.001, ****p < 0.0001) (Scale bars: 50 µm).

Both microglia and astrocytes are key drivers of the inflammatory response following brain injury, with the associated alterations in morphology being a hallmark to this pathway. We and others have reported early and persistent inflammation is associated with glial activation in the FGR brain^17,18,37,40,41^. We examined if the administration of cECFC modulates the pro-inflammatory environment of the FGR brain. Labelling with the microglia marker Iba-1 revealed microglia in the parietal cortex of NG brain that displayed characteristic ramified (resting) morphology, with long fine process extensions and even distribution across the cortex (Fig. 5A). In the FGR brain, microglia displayed enlarged cell bodies and thickened retracted processes as previously reported (Fig. 5A)^17,37^ with a significant increase in the number of total microglia (*p* = 0.0007) and activated microglia (*p* < 0.0001) in FGR when compared with NG (Fig. 5C, D, respectively).

Treatment with cECFC in FGR resulted in microglial morphology comparable to NG (resting state) with significantly lower numbers of total microglia (*p* < 0.0001) and activated microglia (*p* < 0.0001) compared with FGR brain (Fig. 5C, D, respectively). Microglia were also examined in white matter regions including the intragyral (IGWM), subcortical (SCWM), and periventricular white matter (PVWM) (Supp. Table 2, Supp. Fig. 2A, B). Similar findings were observed with FGR displaying increased activation of microglia relative to NG, which was largely ameliorated in the cECFC-treated FGR animals (Supp. Fig. 2A, C). No significant difference in Iba-1-positive activated microglia was evident in PVWM between cECFC-treated and untreated FGR animals (Supp. Fig. 2C). Given our cECFC preparation contains MSCs and previous studies have described the anti-inflammatory properties of MSC cells we also administered MSCs alone as a comparison treatment group. MSCs alone demonstrated similar potency in the cortex, with largely normalised microglial morphology and a significant reduction in the number of activated microglia (Supp. Fig. 3A, B).

We next examined astrocytes, which are involved in maintaining homoeostasis of the CNS, promoting neuronal development, and responding to insults. In the NG pig brain GFAP-positive astrocytes demonstrated multiple long branching processes from the cell body typical of normal astrocyte morphology and were evenly dispersed across the cortex (Fig. 5E). In comparison, astrocytes in the FGR brain displayed reactive morphology, with retraction and thickening of processes into the cell body (Fig. 5E) as previously demonstrated^17^. We observed an increase in the number of GFAP-positive cells (*p* = 0.019; Fig. 5F) compared with NG indicating astrogliosis in the FGR brain. GFAP-positive astrocyte density was also significantly increased in the FGR cortex suggesting an increase in reactive astrocyte morphology (*p* = 0.006; Fig. 5G), IGWM, SCWM and PVWM (Supp Fig. 2D, E) as previously demonstrated^17^.

Both cECFC and MSC-only treated FGR groups presented ramified astrocytic morphology comparable to that observed in NG animals. cECFC-treated FGR animals displayed significantly lower GFAP-positive astrocyte density in the cortex (*p* = 0.046; Fig. 5G), IGWM and PVWM (Supp. Table 2, Supp. Fig. 2E), as well as lower cell counts (*p* = 0.020; Fig. 5F) compared with FGR. Treatment with MSCs alone did not significantly reduce GFAP positive labelling compared with FGR (Supp. Fig. 3C, D). Together these findings suggest an anti-inflammatory effect modulated by MSCs but that combination with ECFC–cECFCs is more efficacious.

Using polymerase chain reaction (PCR) arrays of chemokines and cytokines, we report altered expression of both pro- and anti-inflammatory genes in the FGR cortex compared with NG brains (Fig. 5H). Critical mediators of a pro-inflammatory response including CCL2, CXCL10, IL-1β and TNFα were upregulated in FGR compared with NG (Fig. 5H, I). These cytokines are expressed in glial and neuronal cells in the FGR brain at postnatal day 4^17^ and likely contribute to the ongoing pro-inflammatory cycle. cECFC-treated FGR animals showed reductions in pro-inflammatory genes, specifically key mediators such as IL-1β, TNFα, and CXCL10 (Fig. 5H- J). Anti-inflammatory mediators IL-4, IL9 and TGF-β2 showed lower expression in FGR and were upregulated following cECFC treatment in FGR animals (Fig. 5H, K, L). Our findings indicate a modulation of inflammatory mediators rather than suppression or complete cessation following cell administration. Together with the reduction in overt glial activation, it is likely the inflammatory environment is more precisely regulated in cECFC-treated FGR brains when compared with FGR.

### cECFC treatment reduces neuronal apoptosis in the FGR brain

**Fig. 6:**
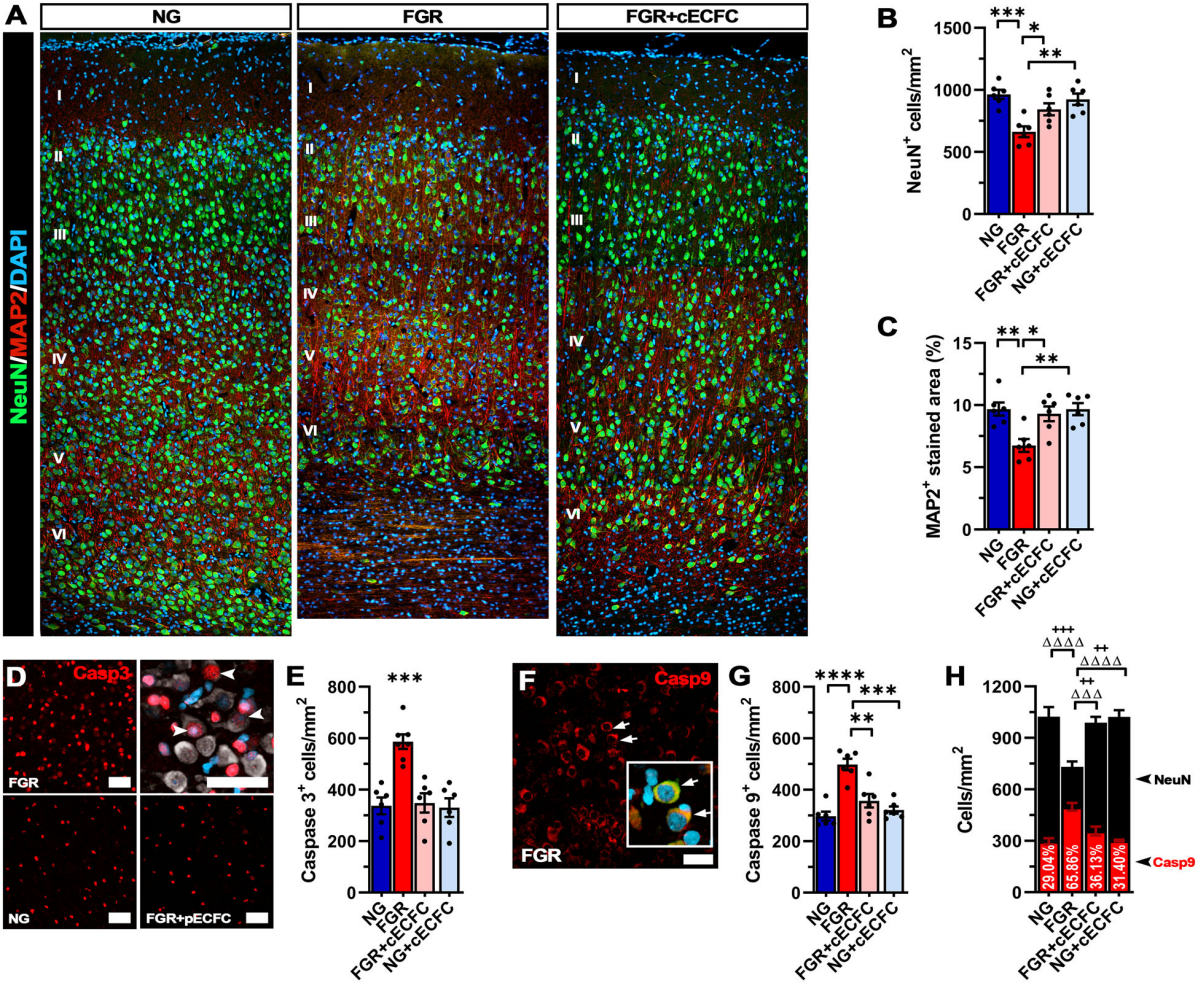
Neuronal integrity is recovered in FGR cortex following cECFC treatment. **A** Representative labelling of neurons in the cortex at day 4. NG demonstrates dense labelling of mature neurons (NeuN; green) and microtubule-associated protein 2 (MAP2; red). FGR demonstrates altered labelling for both NeuN (green) and MAP2 (red). Neuronal labelling is sparse and MAP2 labelling showed weaker perikarya and dendritic labelling compared with other groups. These observations were evident across most cortical layers (II-VI). **B** Quantification of labelling demonstrated decreased numbers of NeuN-positive cells in FGR cortex which was not observed in FGR + cECFC brain. **C** MAP2 quantification also demonstrated a similar trend with FGR + cECFC displaying values similar to NG cortex. **D** FGR cortex demonstrated high numbers of casp3-positive cells, with limited co-localisation to mature neurons (NeuN; grey). **E** FGR displayed elevated cleaved-casp3-positive cells relative to all other groups. **F** Labelling for the initiator caspase (Caspase 9; red) showed clear localisation in mature neurons (see insert: co-localisation with NeuN indicated with arrows). **G** Quantification of casp9-positive cells showed an increase in FGR relative to all other groups. **H** Co-localisation analysis of casp9^+^/NeuN^+^ cells demonstrated significantly increased proportion of neurons undergoing initiation of apoptosis in FGR cortex relative to all other groups (+ denotes significance between neuronal counts, Δ significance between casp9 counts). All values are expressed as mean ± SEM (minimum *n* = 6 for all groups). Two-way *ANOVA* with Tukey post-hoc test (*p < 0.05, **p < 0.01, ***p < 0.001, ****p < 0.0001) (Scale bars: 50 µm).

We have previously reported that FGR results in significant reductions in the number of NeuN-positive neuronal cells in the parietal cortex compared with NG at postnatal day 4^17^. FGR cortex demonstrated regions sparse in neurons, as labelled with the neuronal nuclei marker (NeuN) and the structural neuronal marker (MAP2) (Fig. 6A). In contrast, cECFC-treated FGR brains showed densely packed neurons well distributed and organised into the cortical layers recapitulating observations in NG animals (Fig. 6A). Quantification of labelling showed a significant reduction in NeuN-positive cells in FGR compared with NG (*p* = 0.0005; Fig. 6B, Supp. Table 2). MAP2 labelling was also decreased in FGR compared with NG (*p* = 0.005; Fig. 6C). Following cECFC treatment in FGR, there was a recovery in the number of NeuN-positive cells (*p* = 0.039) and MAP2-positive labelling resembled that observed in NG animals (*p* = 0.015, Supp. Table 2).

We have previously demonstrated an increase in cellular apoptosis in the FGR brain and the ability of the anti-inflammatory ibuprofen to ameliorate this increase^17,37^. We therefore proceeded to determine whether administration of cECFC could also modulate apoptotic activity in the FGR brain.

FGR brain showed a significantly higher number of cells positive for cleaved caspase-3 (Casp3) compared with NG brain (*p* < 0.001; Fig. 6D, E). Administration of cECFCs significantly reduced the number of Casp3-positive cells in the parietal cortex compared with untreated FGR animals (*p* < 0.001; Fig. 6D, E, Supp. Table 2). Low co-localisation of Casp3 and NeuN-positive cells was observed across all brains examined. We, therefore, investigated whether the neuronal cell bodies are undergoing early initiation of apoptosis using caspase-9 (Casp9). We observed a significant increase in Casp9-positive cells in FGR compared with NG (*p* < 0.001; Fig. 6F, G), which was ameliorated following cECFC treatment (*p* = 0.002; Fig. 6F, G, Supp. Table 2). In those cells that labelled with Casp9, 65% were found to co-label with NeuN (*p* < 0.0001) in FGR compared with only 29% in NG animals, suggesting that neuronal cells have been flagged to undergo apoptosis (Fig. 6H). cECFC treatment significantly reduced the proportion of Casp9-positive apoptotic neurons in the FGR-treated animals compared with FGR animals. There were no significant differences in Casp9-positive cell counts between NG animals and NG cECFC-treated (*p* = 0.846) animals (*p* = 0.201).

### cECFC treatment ameliorates white matter disruption in the FGR brain

**Fig. 7:**
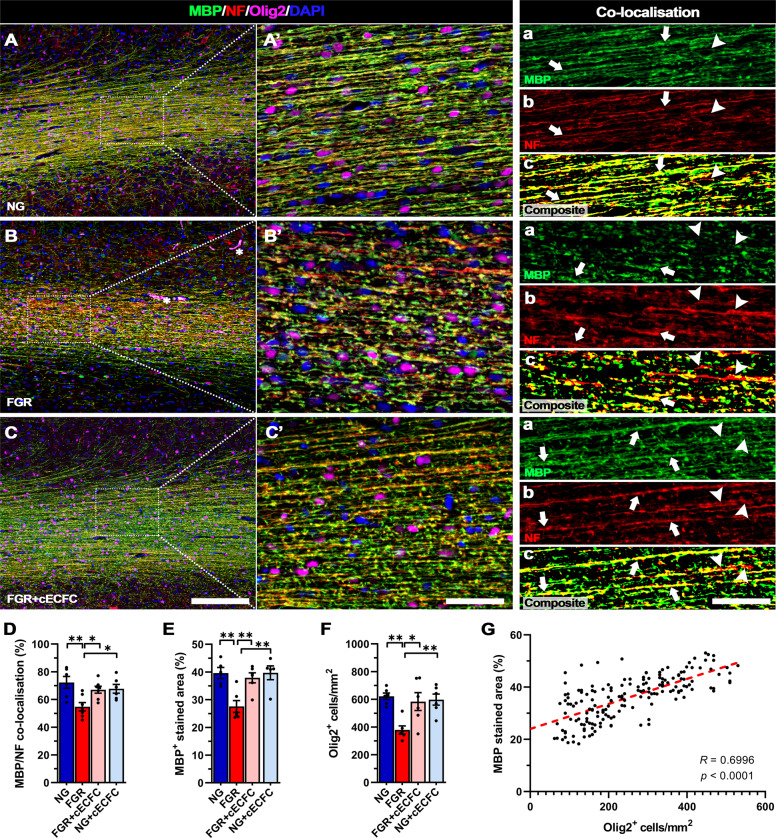
Improved axonal myelination following cECFC treatment in FGR. Representative labelling of myelin (MBP), neurofilament (NF) and pan-oligodendrocyte (Olig2) expression in white matter of NG, FGR and FGR + cECFC brains at postnatal day 4. **A** NG showed robust labelling for each marker with consistent presence of Olig2-positive cells along fibres. MBP and NF displayed strong co-localisation and structure throughout the white matter (**A’a-A’c**; arrows). **B** FGR display disrupted labelling of MBP and NF, with decreased Olig2-positive cells. There was observable disruption in labelling patterns with an evident loss in co-localisation of MBP to NF (**B’**; arrowheads). **C** FGR + cECFC brains showed normalised labelling and organisation of MBP and NF comparable that observed in NG. **D** Analysis of MBP/NF co-localisation in white matter shows a loss in axonal myelination in FGR compared with all groups. **E** Quantification of MBP-positive stained area (%) showed significantly decreased coverage and **F** decreased Olig2-positive cells/mm^2^ in FGR white matter relative to all groups, both of which were largely ameliorated following cECFC treatment. **G** Correlative analysis demonstrates a positive relationship between Olig2-positive cell count and MBP-positive stained area. All values are expressed as mean ± SEM (minimum *n* = 6 for all groups). Two-way ANOVA with Tukey post-hoc test (*p < 0.05, **p < 0.01, ***p < 0.001) (Scale bars: **A**–**C**: 200 µm; **A’**–**C’** and **a**–**c**: 50 µm).

FGR is associated with significant alterations in white matter structure and organisation^42,43^. Our previous studies have reported decreased myelination and impaired myelination based on Luxol fast blue staining of the white matter in FGR animals at postnatal day 1 and 4^17,37^. Here, we investigated expression of myelin binding protein (MBP), neurofilament (NF), and the pan-oligodendrocyte marker (Olig2) in white matter. NG brains demonstrated well organised and consistent labelling for each of these markers along lengths of the white matter tracts, with strong MBP and NF co-localisation (72.3% co-localisation; Fig. 7A-Ac, D). In comparison, FGR white matter presented more truncated and uneven labelling across the length of the white matter tracts for both MBP and NF (Fig. 7B, B’). Analysis showed a significant loss in MBP-positive labelling in the white matter of FGR compared with NG (*p* = 0.004; Fig. 7E). Co-localisation analysis found evidence of NF-positive axons displaying minimal to no MBP labelling, indicating partial loss of myelination along the axonal length (FGR: 54.8% co-localisation; Fig. 7Ba-c, D). cECFC-treated FGR animals displayed similar labelling patterns to those observed in NG brain (Fig. 7C-Cc, D). cECFC-treated FGR animals displayed significantly higher MBP-positive labelling compared with untreated-FGR animals (*p* = 0.009; Fig. 7E, Supp. Table 2). A decrease in the number of Olig2-positive cells was also observed in FGR animals compared with NG (*p* = 0.003; Fig. 7F) which was restored by cECFC treatment (Fig. 7F). This decrease in Olig2 cell count was attributed to an increase in apoptosis of Olig2-postive cells. Co-labelling of Olig2 with the apoptotic marker cleaved caspase-3 (Casp3), showed a significantly higher percentage of Olig2 cells were undergoing apoptosis in FGR compared with NG (FGR: 51% cf NG: 23%, *p* = 0.0001; Supp Fig. 4A, B). Once again, cECFC-treated FGR showed a reduction in the number of apoptotic Olig2 cells (33%) compared with FGR (*p* = 0.012; Supp Fig. 4A, B). We next examined whether the loss of Olig2-postive cells in the white matter was associated with the degree of MBP labelling. Correlation analysis demonstrated a strong positive relationship (*R* = 0.700, *p* < 0.001; Fig. 7G), of loss of Olig2 cells and lower myelination^17^. White matter tracts in FGR brain displayed more dispersion and varied orientation based on MBP-positive labelling. Analysis of MBP orientation confirmed a higher degree of dispersion in the FGR brain compared with NG brain (*p* = 0.015; Supp Fig. 4C, D). cECFC-treated FGR animals displayed significantly lower WM tract dispersion than FGR animals (*p* = 0.046; Supp Fig. 4C, D). These findings indicate administration of cECFCs promoted Olig2 survival, maintaining axonal myelination and organisation of white matter tracts.

## Discussion

Neurodevelopmental delays occur in 24–53% of FGR infants at 2 years of age^44,45^ and may be a result of neurodevelopmental impairment as a consequence of FGR. There are no treatment options currently available to protect the FGR newborn. This study provides evidence that postnatal intervention of placentally derived cECFC treatment affords neuroprotection in the FGR piglet. We demonstrate cECFC infusion improves vascularisation, NVU integrity, recovery of neuronal maturation and white matter development in the FGR brain. Our findings indicate that cECFC treatment provides neuroprotection in the FGR neonate via targeting both inflammation and the NVU.

In the current and previous study, we observed changes to neurons and glial cells in the postnatal FGR brain^17,37^. Examination of vascular endothelial cells and basement membrane revealed a significant reduction in these cell types suggesting a loss in cerebrovasculature in the FGR brain with a significant decrease in total vascular length and vessel branching in the FGR brain.

MSC only treatment had no significant effect on improving the vasculature in the FGR brain. However, cECFC treatment not only increased vessel density it also improved vascular length in the FGR brain. cECFC treatment did not show a significant difference in vessel branching suggesting cECFC treatment does not promote unwarranted angiogenesis. These findings support the potential of cECFCs to improve cerebrovascularisation in the FGR neonate. On the contrary to our findings of reduced endothelial cells in FGR brain, an increase in endothelial cells (GLUT-1) was reported in both FGR and FGR UCB treated lambs in comparison to normally grown lambs^24^. This discrepancy may be due to the marker used to label the endothelial cells. Yet Grandvuillemin et al. showed improved capillary density (eNOS) follow both umbilical cord blood (UCB) and ECFC treatment at 7 days and 12 weeks after treatment in neonatal hypoxic-ischaemic encephalopathy (HIE) rat model^46^. The authors also showed an increase in cerebral blood flow at 12 weeks for both treatments demonstrating evident functional improvement to the cerebral vasculature.

There was evidence of BBB disruption in the piglet FGR brain, with endogenous proteins albumin located in the perivascular space and extravasation of IgG into the brain parenchyma. Juxtavascular astrocytes displayed activated morphology including hypertropic end-feet, and in some instances we found absence of vessel–glial contact. cECFC treatment improved BBB integrity with low-intensity albumin labelling restricted to the vessel lumen and juxtavascular astrocytes reverting to a ramified astrocyte morphology. Even though cECFC treatment did not significantly reduce the percentage of vessels with perivascular albumin labelling it did significantly reduce albumin labelled area compared with FGR. In a lamb FGR model, albumin was shown to enter the tissue parenchyma which we did not observe in our current study, however, the authors also showed a reduction in albumin extravasation following UCB treatment supporting the effect of stem cells on BBB integrity^24^.

Impaired astrocyte end–feet interaction with neurovasculature may be associated with altered NVU integrity in the FGR brain^24^. Following cECFC treatment, the FGR piglets displayed similar characteristics to NG animals with an abundance of juxtavascular astrocytes in consistent contact along the vasculature. This combination of ECFCs and MSCs may be working together to stabilise the NVU and by reducing the pro-inflammatory environment which in turn results in juxtavascular astrocytes returning to their normal function at the NVU. In the FGR lamb, while UCB treatment did not alter astrogliosis, the authors observed an increased association of smooth muscle proteins of the basal lamina with pericytes in the NVU^24^.

Clinical and animal studies provide evidence that inflammation is perpetuated long after birth, providing the opportunity of targeting inflammation soon after birth which may be critical to reducing detrimental inflammatory events and subsequent injury to the FGR brain^47,48^. We and others have reported that early and persistent inflammation in the brain is associated with glial activation in the FGR neonate^14,17,24,37^. In the current study we have demonstrated an overt glial response in the FGR piglet brain after birth with an increase in activated microglia and astrocytes in both the grey and white matter. Microglia responded to both MSC only treatment and cECFC treatment with a decrease in both the number and activation of microglia throughout the brain parenchyma. However, cECFC treatment was more efficacious than treatment with MSC alone at minimising astrocyte activation in the FGR piglet brain. The MSC alone treatment results are corroborated by an FGR lamb study^24^. The authors demonstrated when allogeneic UCB mononuclear cells (25 million/kg) was administered 1 h after birth, activated microglial cells were reduced but not astrocytes in the white matter 24 h post-treatment^24^. An FGR rat study also showed that umbilical cord-derived mesenchymal stromal cells (UC-MSC) (1 × 10^5^) did not affect the number of astrocytes 7 days after treatment^49^. Astrocytes have many functions in the brain and are located in the brain parenchyma as well as at the NVU. The positive response of glia in the tissue parenchyma we observe following cECFC treatment may be due to restoration of cerebrovascularisation and NVU integrity. As the combination of ECFCs and MSCs are stabilising the NVU and increasing the anti-inflammatory state (discussed below), these interrupt the perpetual inflammatory environment in the brain. However, one study showed a similar inflammatory response between UBC and ECFC treatment in a neonatal HIE rat model^46^. This study administered stem cells intraperitoneally 48 h after insult—10^5^ ECFC or 10^7^ UCB. At 7 days post treatment, they demonstrated a reduction in neuroinflammation (iNOS) after both treatments, but no change in astrocyte numbers. However, at 12 weeks a significant reduction in GFAP-positive cells was reported in both groups demonstrating a delayed decrease in astrogliosis. Demonstrating long-term studies are necessary to determine the longevity of treatment effects.

Increased glial cell activation is associated with a pro-inflammatory environment in the FGR brain^47^. The current study concurs with previous reports showing an increase in pro-inflammatory cytokines in the FGR brain and an altered anti-inflammatory profile^17,37^. We have previously demonstrated anti-inflammatory treatment (ibuprofen) in the FGR newborn piglet reduces levels of pro-inflammatory cytokines^17^, however in the current study cECFC treatment exerted a greater effect on increasing anti-inflammatory cytokines. The prominent increases in anti-inflammatory cytokines may be due to reprograming of the microglia into an anti-inflammatory state. Recent in vitro studies suggest that a key therapeutic function of MSCs is their ability to reprogramme brain microglia into an anti-inflammatory state characterised by increased phagocytic activity and upregulated expression of anti-inflammatory mediators, which in turn contributes to reduced inflammation and promotes tissue repair^50^. These findings are corroborated in an FGR rat study examining UC-MSC treatment^49^. The authors demonstrate no significant effect of treatment on pro-inflammatory microglia however anti-inflammatory microglia were significantly increased in the treatment group compared with sham, demonstrating an anti-inflammatory effect of stem cell treatment in the FGR rat. An FGR lamb study reported a reduction in the pro-inflammatory cytokine TNFα following UCB treatment, similar to the response we demonstrated following cECFC treatment. However, they report no effect of UCB on any other pro-inflammatory cytokines measured^24^. This finding is further supported by recent study which has shown the ability of ECFCs in suppressing T-cell proliferation and programming toward less pro-inflammatory phenotypes. The study demonstrated the significance of the TNFα/TNFR2 pathway in mediating this response^51^. Recent study has shown that priming of ECFCs with TNFα (1 ng/mL) prior to administration was able to further enhance the immunosuppressive phenotype, potentially resulting in prolonged presence in vivo to enhance regeneration^52^. We demonstrated that cECFCs have a combined effect of reducing pro-inflammatory cytokines while concurrently increasing anti-inflammatory cytokines. MSCs and ECFCs target different cell populations to maintain a healthy brain environment, and therefore this multicellular effect may be the key to modulating the inflammatory state in the brain.

Clinical imaging studies describe persistent grey and white matter disruption in the human FGR neonate that are associated with developmental disabilities at 1 year of age^42,53^. Ongoing neuronal and white matter impairment is also evident in the FGR piglet brain after birth at a microscopic level^17,36^. In the current study, we saw regions sparse in neurons (mature and structural neurons) and disruption to mature myelin and oligodendrocytes throughout the white matter in the FGR brain. Inflammation is a key driver of neuronal and white matter injury and when postnatally targeted with an anti-inflammatory intervention as we have done here with cECFCs and previously with the anti-inflammatory ibuprofen, a corresponding reduction in neuronal and white matter disruption is evident^17^.

The loss in neurons observed in the FGR brain may be attributed to pro-apoptotic events as a result of the pro-inflammatory environment. We reported a significant increase in the early apoptotic initiator, caspase-9 in neurons in the FGR brain. We have previously demonstrated an increase in cellular apoptosis and the ability of the anti-inflammatory ibuprofen to ameliorate this increase^37^. cECFC treatment reduced neuronal cells that labelled for both caspase-3 and caspase-9. The modulation of the inflammatory response following stem cell administration likely contributed, either directly or indirectly, to the reduced apoptotic activity in the FGR brain, An HI rat study reported similar reduction in apoptotic cells (TUNEL) and concurrent recovery to neuronal cell counts 7 days after treatment with either UCB or ECFCs^46^. This recovery to neurons could be due to reversal of apoptosis or reinstatement of normal development.

We show not only disruption to myelination as previously demonstrated^17,36^, but also disruption to oligodendrocytes as observed in other animal models of FGR^15,18,54,55^. We demonstrate partial loss of myelination along the axon in the FGR brain with increased apoptotic oligodendrocytes. White matter tracts in the FGR brain displayed more dispersion and varied orientation suggesting that loss of oligodendrocytes in FGR results in decreased and disorganised myelination of white matter tracts which likely contributes to the long-term white matter alterations reported in the FGR brain^56^. Administration of cECFC treatment promoted oligodendrocyte survival, maintaining axonal myelination and organisation of the white matter tracts. Only one other study has examined white matter response to stem cell treatment in the FGR newborn. This study in the FGR rat showed no difference in white matter volume following UC-MSC treatment^49^. However, this FGR model may not be appropriate to study FGR with no detectable white matter injury. White matter injury is a hallmark feature of FGR^57^ as such there should be noticeable white matter changes in any FGR animal model. In the current study, we demonstrate recovery to both grey and white matter cellular impairment within the parietal cortex following cECFC treatment in the FGR piglet brain.

The developmental progression of FGR brain injury likely occurs after 27 weeks gestation as reported in a human study^58^. A similar response is observed in the FGR piglet where significant brain injury is observed at 104 days gestation (equivalent to 26–28 weeks human gestations^59^) but not prior^36^, demonstrating this brain injury commences during the third trimester. As this injury occurs later in gestation and a large percentage of FGR fetuses are not detected until the time of birth, stem cell treatment around the time of birth is a feasible and appropriate option to protect the FGR newborn from sustained brain injury.

The use of postmortem histological examination allows for in-depth analysis of key structural changes contributing to neuropathology, however, there are limitations with the range of phenomena one can observe. In the present study, we utilised several markers to interrogate interactions between cell types following cECFC treatment in the FGR brain, however, these do not allow mechanistic insight of cell-cell communication. This limitation is highlighted by our inability to separate vascular and neuronal effects of the cECFC treatment. It is unknown whether the benefits we observed following treatment are a direct consequence of increased vessel density and reduced BBB permeability or whether the cECFCs directly communicate with other cells such as astrocytes or microglia. Further in vitro studies would address this limitation. We observed no sex-related differences in any of the analyses undertaken. However, another limitation of small animal numbers in the current study make it difficult to fully discern sex differences.

MSC therapy alone has variable neuroprotective effects through modulation of inflammation in small animal models of cerebral ischaemia^60–62^. However, with the addition of ECFCs to aid in vascular regeneration, this enhanced stem cell preparation has the potential to significantly improve brain outcomes for FGR neonates. Previous studies have shown cECFCs are not rejected in immunocompetent animal studies^29^ demonstrating important findings where these cells can be used in allogeneic situations. The use of healthy placentas and in an off-the-shelf allogenic scenario would ensure continued availability and provide a more appropriate solution than using autologous cells from a pathologic FGR placenta^63^.

We regard cECFC treatment as a promising multicellular approach to protecting the FGR newborn brain. This combination of fetally derived stem cells improved the brain microenvironment only 3 days after treatment in the FGR newborn piglet. Future preclinical studies to confirm long-term efficacy and safety are essential in the success of human clinical trials for stem cell treatment in the FGR newborn.

## Methods

### Animals

Approval for this study was granted by The University of Queensland Animal Ethics Committee (UQCCR/420/17) and was carried out with respect to the National Health and Medical Research Council guidelines (Australia) and ARRIVE guidelines. Animals care was in accordance with institutional guidelines.

Newborn Large White piglets (*n* = 36; <18 h) were collected from the UQ Gatton Piggery monitored and cared for at the Herston Medical Research Centre until day of euthanasia on postnatal day 4 (P4). Litter matched pairs were obtained from multiple sows (*n* = 14 litters). Piglets were divided into five experimental groups with pigs randomly assigned to treatment groups: NG (*n* = 8), FGR (*n* = 8), FGR + cECFC (*n* = 8); FGR + MSC (*n* = 5), and NG + cECFC (*n* = 7); with equal males and females in each group. FGR piglets were defined by birth bodyweight (<10th percentile on the day of birth) and confirmed through brain to liver weight ratio (B:L) ≥ 1 assessed post-mortem at P4^36,64,65^.

On P4, piglets were euthanised via an intraperitoneal injection of sodium phenobarbital (650 mg/kg; Lethabarb, Virbac, Australia). Brains were transcardially flushed with phosphate-buffered saline, and brain tissue was collected, weighed, hemisected and coronally sliced. The right hemisphere sections were immersion fixed in 4% paraformaldehyde as previously described^66^. The parietal cortex from the left hemisphere was snap frozen in liquid nitrogen and stored at −80 °C for mRNA analysis.

### Fluorescence-activated cell sorting

Primary conjugated antibodies were used for fluorescence-activated cell sorting (FACS). Placental tissues were processed, and single-cell suspension was prepared as reported previously^32,35^. The isolated placental CD34+ single-cell suspension was incubated with human CD34-phycoerythrin (PE) (Bio-Rad; dilution 1:25), human CD45-FITC (BioLegend; dilution 1:25) and human CD31-V450 BD Biosciences: dilution 1:30) for 20 min at 4 °C. Cells were flow sorted using FACSAria Fusion (Becton Dickinson). Cell doublets were removed and 7-amino-actinomycin D (BD Pharmingen; dilution 1:40) was used to exclude dead cells. Fluorescence minus one (FMO) control was used in gating the population of interest. To remove any remaining contaminating CD45^+^ cells from the hematopoietic lineage, only CD45^-^CD34^+Hi^ population was gated and further sorted according to their level of CD31 expression. The population of interest, the CD31^-^ and CD31^Int^ populations were sorted directly into 100% foetal bovine serum (FBS; Gibco) before being plated for cell culture.

### Stem cell culture

Human placental foetal ECFC and MSC were isolated using our previously published protocol^32^. ECFC and MSC preparations were sourced from three different donors. Human term placenta were obtained with written informed consent from healthy women undergoing caesarean deliveries at term (38–39 weeks of gestation) at the Royal Brisbane and Women’s Hospital, as approved by both the University of Queensland and the Royal Brisbane and Women’s Hospital human research ethics committees (RBWH HREC/09/QRBW/14 & UQ 2009000508). This approval allows for the isolation and use of any stem cell populations obtained from the placental tissue.

The isolated foetal ECFC and MSC were cultured on rat tail collagen-coated tissue-culture flasks in Endothelial Growth Medium (EGM2) (Lonza) with 10% of FBS. The isolated cells demonstrated similar functional profiles. The foetal ECFC and MSC were characterised via flow cytometry using a suite of cell surface markers to ensure their correct phenotype. The cell surface markers include: CD144, CD31, CD34, CD105, CD44, CD146, HLA-ABC, HLA-DR, HLA-DR/DP/DQ, CD45, CD29, CD73, CD90 and expression was as expected and previously observed^32,35^ (Supp. Fig. 6). Details of antibodies are listed in Supp. Table 1. For in vivo stem cells treatment, 10^6^ ECFC 10^6^ MSC were injected intravenously into NG and FGR piglets (Fig. 1A, B). In a further subset of FGR animals, 10^6^ MSC only were injected. The use of human ECFCs and MSCs over porcine cells allows for the rapid translation of this treatment to clinical trials in humans.

### Magnetic resonance imaging

We used magnetic resonance imaging (MRI) techniques to determine whether we could detect in vivo brain alterations in the FGR piglet at postnatal day 4. Piglets were anesthetised with isoflurane (1–3%) mixed with oxygen (2 L/min) for the duration of the scanning protocol. Each piglet was placed in a 300 mm bore 7T ClinScan MR scanner (Bruker, Germany), running Siemens VB17. A 150 mm ID MRI rf coil was used to acquire the dynamic images of sagittal, coronal and axial slices. Images were obtained at TR/TE 8500/60 ms, 1.6 mm slices thickness, 64 × 64 acquisition matrix. Region of interest over the parietal cortex was defined on the T2 map and applied to the ADC map and raw values extracted.

### Magnetic resonance spectroscopy acquisition and analyses

1H-MR spectroscopy were obtained using the 7T Bruker/Siemens whole-body. A single spectrum was acquired the frontoparietal region of the brain from a 10 mm^3^ voxel using single-voxel spectroscopy with the following parameters: TR = 6000 ms, TE = 60 ms and 128 averages. Metabolite spectra were exported, processed, and analysed using the Advanced Method for Accurate, Robust and Efficient Spectral Fitting tool within jMURI and manually phased^67^. Before quantification, apodisation (10 Hz) was applied to the N-acetyl aspartate (NAA), creatine (Cr) and choline (Cho) peaks with Lorentzian peaks. Peak areas of metabolites were estimated using Lorentzian profiles and a priori knowledge with soft constraints (initial chemical shift, amplitude, and peak widths). NAA was set as a reference at 2 ppm chemical shift. Peak area ratios were calculated for NAA/Lac, NAA/Cho, NAA/Cr, Lac/Cr, Lac/Cho, Lac/NAA and Cho/Cr.

There were no overt differences in brain structure as assessed with T2 and ADC analysis between FGR and NG animals. There were no significant MRS alterations in metabolite levels between NG and FGR. Administration of cECFCs did not result in any noticeable changes when compared with NG and FGR (Supp. Fig. 5).

### Immunohistochemistry

Brain slices containing parietal cortex from the right hemisphere (NG = 8; FGR = 8; FGR + cECFC=6; NG + cECFC=6; FGR + MSC = 5) were embedded in paraffin and sectioned at 6 µm (Pig stereotaxic map, A 5.5 mm;^68^). For vessel structure and glial interaction analyses brain sections of 12 µm thickness were used. Sections were affixed to Menzel Superfrost Plus adhesive slides and dried overnight at 37 °C. All sections were dewaxed and rehydrated using standard protocols followed by heat-induced epitope retrieval with 10 mM citrate buffer (pH 6) or TRIS-EDTA buffer (pH 9) at 90 °C for 20 min before cooling to room temperature (RT). Sections were blocked with 5% donkey serum in PBS with 0.5% Triton-X 100 for 1 h at RT. Primary antibodies (Supp. Table 1) were incubated overnight at 4 °C. Slides were washed in tris-buffered saline followed by incubation with species-specific secondary fluorophores (Supp. Table 1) at RT for 1 h. Sections were washed, counterstained with 4′,6-diamidino-2-phenylindole, and mounted with Prolong Gold antifade (Molecular Probes, Invitrogen Australia, Victoria, Australia). Negative control sections without primary antibodies were processed in parallel and immunolabelling was conducted in triplicates for all animals.

### Image acquisition and analysis

Acquisition of immunolabelled sections was performed using a Zeiss Axio Microscope fitted with an Axiocam503 camera and ZEN 2012 software, using either an EC Plan-Neofluar 20x/0.50 M27 (FWD = 2.0 mm) or EC Plan-Neofluar 40x/0.75 M27 (FWD = 0.71 mm) objective. Four pictomicrographs (16-bit; 1936 × 1456) of the parietal cortex and white matter regions were captured for analysis from each section. For all markers, replicates were conducted with sections separated by at least 70 µm. All imaging and analyses were conducted under blind conditions (KKC, JAW, ET and LJ). Microglia were manually counted and categorised with respect to morphology as per previous studies^17,37^. Density analysis, co-localisation and vessel coverage analyses were undertaken using the threshold function with moments plugin in FIJI (ImageJ; Image Processing and Analysis in Java; National Institutes of Health, Bethesda, MD, USA).

### Quantitative polymerase chain reaction (qPCR)

qPCR was conducted as previously reported^17,37^. Total RNA from frozen parietal cortex samples was isolated using RNeasy Tissue Mini Kit (Qiagen). Yield and quality were determined using a NanoDrop spectrophotometer (ND-1000 system). Reverse transcription kits (RT2 First Strand Kit; Qiagen) were used for cDNA synthesis. cDNA was pooled for each experimental group giving equal concentrations from every animal in the pooled sample. Pooled cDNA was combined with RT2 SYBR Green qPCR Mastermix (Qiagen) and loaded into the Pig Inflammatory Cytokines & Receptors RT2 Profiler^TM^ PCR Array (Qiagen, Hilden, Germany). The qPCR reactions were performed using a Qiagen Rotor-Gene Q real-time cycler. The amplified transcripts were quantified with the comparative CT method using actin, gamma 1 (ACTG1) mRNA expression levels for normalisation. The same CT threshold value was used across all arrays to allow comparison between runs. Each experimental group was run across two arrays with samples randomly assigned to each array.

### Statistics

Unless otherwise specified, Two-way ANOVA with the Tukey post-hoc analyses was used to determine differences between NG and FGR animals under non-treated and stem cell treated conditions (Graph Pad Prism 9.0 software, San Diego, California, USA). Results are expressed as mean ± SEM with statistical significance accepted at *p* < 0.05. Indicated sample sizes (n) represent biological replicates including individual samples and have been listed in the figure legends where appropriate.

### Reporting summary

Further information on research design is available in the Nature Research Reporting Summary linked to this article.

## Supplementary information

Supplementary Information

Reporting Summary

## Data Availability

The data that support the findings of this study are available from the corresponding author upon reasonable request.
