# Supplementary material for: Combination of human endothelial colony-forming cells and mesenchymal stromal cells exert neuroprotective effects in the growth-restricted newborn
Source: NPJ Regen Med. 2021 Nov 18;6:75. doi: 10.1038/s41536-021-00185-5 (PMC8602245; doi:10.1038/s41536-021-00185-5)
Supplement: Supplementary file 1 — Supplementary Information [file 41536_2021_185_MOESM1_ESM.pdf]

Supplementary Table 1:

**Primary Antibody**	**Host**	**Dilution**	**Catalogue#**
Anti-human CD34-PE	Mouse	1:25	Bio-Rad (MCA1578PE)
Anti-human CD45-FITC	Mouse	1:25	BioLegend (304006)
Anti-human CD31-V450	Mouse	1:30	BD Biosciences (561653)
Anti-human CD105-PE	Mouse	1:50	BD Biosciences (560839)
Anti-human CD144-FITC	Mouse	1:50	BD Biosciences (560411)
Anti-Human CD90-PE.CY5	Mouse	1:800	BD Biosciences (555597)
Anti-Human CD44-FITC	Mouse	1:50	BD Biosciences (347943)
Anti-Human CD29-BV480	Mouse	1:50	BD Biosciences (746592)
Anti-Human CD73-FITC	Mouse	1:50	BD Biosciences (561254)
Anti-Human CD146-FITC	Mouse	1:50	BD Biosciences (560846)
Anti-Human HLA-ABC-PE	Mouse	1:50	BD Biosciences (565291)
Anti-Human HLA-DR-PE	Mouse	1:50	BD Biosciences (567054)
Anti-Human HLA-DR/DP/DQ-FITC	Mouse	1:50	BD Biosciences (555558)
7AAD		1:40	BD Pharmingen (559925)
Lamin A/C	Rabbit	1:200	Abcam (ab108595)
Endothelial progenitor cells (CD34)	Goat	1:1000	R&D Systems (AF3890)
Collagen IV (Col IV)	Rabbit	1:1000	Abcam (ab6586)
Endothelial cells (CD31)	Rabbit	1:500	Abcam (ab28364)
Albumin	Sheep	1:1000	Abcam (ab8940)
IgG	Goat	1:1000	JIR (114-005-003)
S100B	Rabbit	1:1000	Invitrogen (PA5-78161)
Ionised calcium binding adaptor molecule-1 (Iba-1)	Goat	1:1000	Abcam (ab5076)
Glial fibrillary acidic protein (GFAP)	Mouse	1:1000	Sigma (G3893)
Glial fibrillary acidic protein (GFAP)	Rabbit	1:2000	DAKO (Z0334)
Neuronal Nuclei (NeuN)	Rabbit	1:1000	Abcam (ab177487)
Microtubule-associated protein 2 (MAP2)	Mouse	1:500	Sigma-Aldrich (M4403)
Cleaved Caspase-3	Rabbit	1:500	Cell Signaling (#9661)
Caspase-9	Rabbit	1:500	Abcam (ab32539)
Myelin Binding Protein (MBP)	Rat	1:1000	Abcam (ab7349)
Neurofilament (NF)	Mouse	1:500	Abcam (ab134306)
Oligodendrocyte marker 2 (Olig2)	Rabbit	1:1000	Genetex (GTX132732)
**Secondary Antibody**			
α-Rat Alexa Fluor 488	Donkey	1:1000	Invitrogen (A-21208)
α-Rabbit Alexa Fluor 488	Donkey	1:1000	Invitrogen (A-21206)
α-Goat Alexa Fluor 568	Donkey	1:1000	Invitrogen (A-11057)
α-Mouse Alexa Fluor 568	Donkey	1:1000	Invitrogen (A10037)
α-Rabbit Alexa Fluor 594	Donkey	1:1000	Invitrogen (A-21207)
α-Goat Alexa Fluor 647	Donkey	1:1000	Invitrogen (A-21447)
α-Mouse Alexa Fluor 647	Donkey	1:1000	Invitrogen (A-31571)
DAPI (4',6-Diamidino-2-Phenylindole, Dihydrochloride)	-	1:2000	Invitrogen (D1306)

Supplementary Table 2:

**Glial characterisation**				
Parietal cortex	**NG**	**FGR**	**FGR+cECFC**	**NG+cECFC**
Total microglia (Iba-1$^+$ cells/mm^2)	450.0 ±15.22	602.7 ±26.95 [a,b,c]	497.8 ±16.85	460.3 ±20.14
Activated microglia (Iba-1$^+$ cells/mm^2)	70.32 ±9.52	132.8 ±7.79 [a,b,c]	91.69 ±5.21	80.72 ±5.51
GFAP$^+$ labelled area (%)	8.39 ±0.39	10.96 ±0.61 [a,b,c]	9.01 ±0.50	8.63 ±0.38
GFAP$^+$ cells/mm^2	589.9 ±30.49	775.5 ±45.00 [a,b]	598.8 ±33.91	544.3 ±49.67
S100b$^+$ cells/mm^2	380.4 ±26.38	268.4 ±21.16 [a,b,c]	350.7 ±20.47	367.0 ±18.36
Intragyral white matter				
Activated microglia (Iba-1$^+$ cells/mm^2)	71.7 ±7.63	140.06 ±11.04 [a,b,c]	97.88 ±8.69	74.95 ±6.08
GFAP$^+$ labelled area (%)	6.33 ±0.46	11.15 ±0.55 [a,b]	7.69 ±0.53	6.38 ±0.32
Subcortical white matter				
Activated microglia (Iba-1$^+$ cells/mm^2)	81.42 ±6.87	142.03 ±13.33 [a,b,c]	103.22 ±4.82	82.97 ±4.26
GFAP$^+$ labelled area (%)	6.44 ±0.39	9.92 ±0.62 [a]	8.23 ±0.70	6.28 ±0.26
Periventricular white matter				
Activated microglia (Iba-1$^+$ cells/mm^2)	79.17 ±9.24	136.41 ±16.13 [a,c]	102.24 ±7.99	80.58 ±5.60
GFAP$^+$ labelled area (%)	7.51 ±0.70	11.19 ±0.37 [a,b]	8.81 ±0.48	6.83 ±0.54
**Neuronal and white matter assessment**				
NeuN$^+$ cells/mm^2	964.6 ±36.58	663.3 ±43.27 [a,b,c]	843.8 ±47.93	924.6 ±46.23
MAP2$^+$ labelled area (%)	9.68 ±0.53	6.75 ±0.52 [a,b,c]	9.30 ±0.59	9.67 ±0.49
Caspase 3$^+$ cells/mm^2	337.2 ±32.69	586.4 ±28.30 [a,b,c]	348.2 ±37.71	330.5 ±36.24
Caspase 9$^+$ cells/mm^2	297.5 ±17.26	498.3 ±22.31 [a,b,c]	357.1 ±26.36	321.1 ±14.14
MBP/NF co-localisation (%)	72.31 ±4.30	54.75 ±2.99 [a,b,c]	67.03 ±2.49	67.74 ±3.16
MBP$^+$ labelled area (%)	39.63 ±1.99	27.55 ±2.13 [a,b,c]	37.90 ±1.80	39.71 ± 2.48
Olig2$^+$ cells/mm^2	621.5 ±23.59	378.8 ±29.84 [a,b,c]	583.5 ±64.61	597.9 ±40.25

NOTE:

[a] $p < .05$ vs. NG

[b] $p < .05$ vs. FGR+cECFC

[c] $p < .05$ vs. NG+cECFC

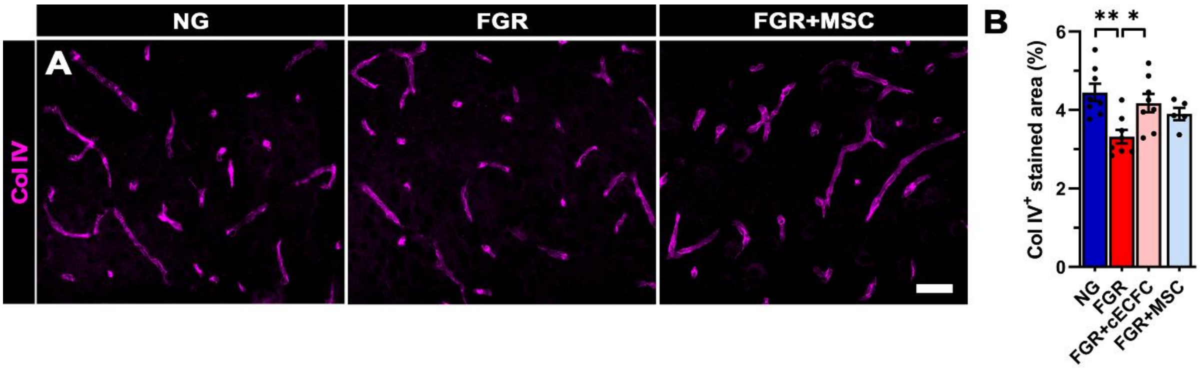

Supplementary Fig. 1. Mesenchymal stromal cell treatment does not improve vascular expression of Col IV in FGR brain. **(*A*)** Col IV labelling showed no significant improvement in microvessel density following treatment with MSC only. **(*B*)** Analysis confirmed no significant recovery in Col IV-positive labelled area. All values are expressed as mean +/- SEM (minimum n=5 for all groups). Two-way *ANOVA* with Tukey post-hoc test (*p<0.05, **p<0.01) (Scale bars: 50 µm)

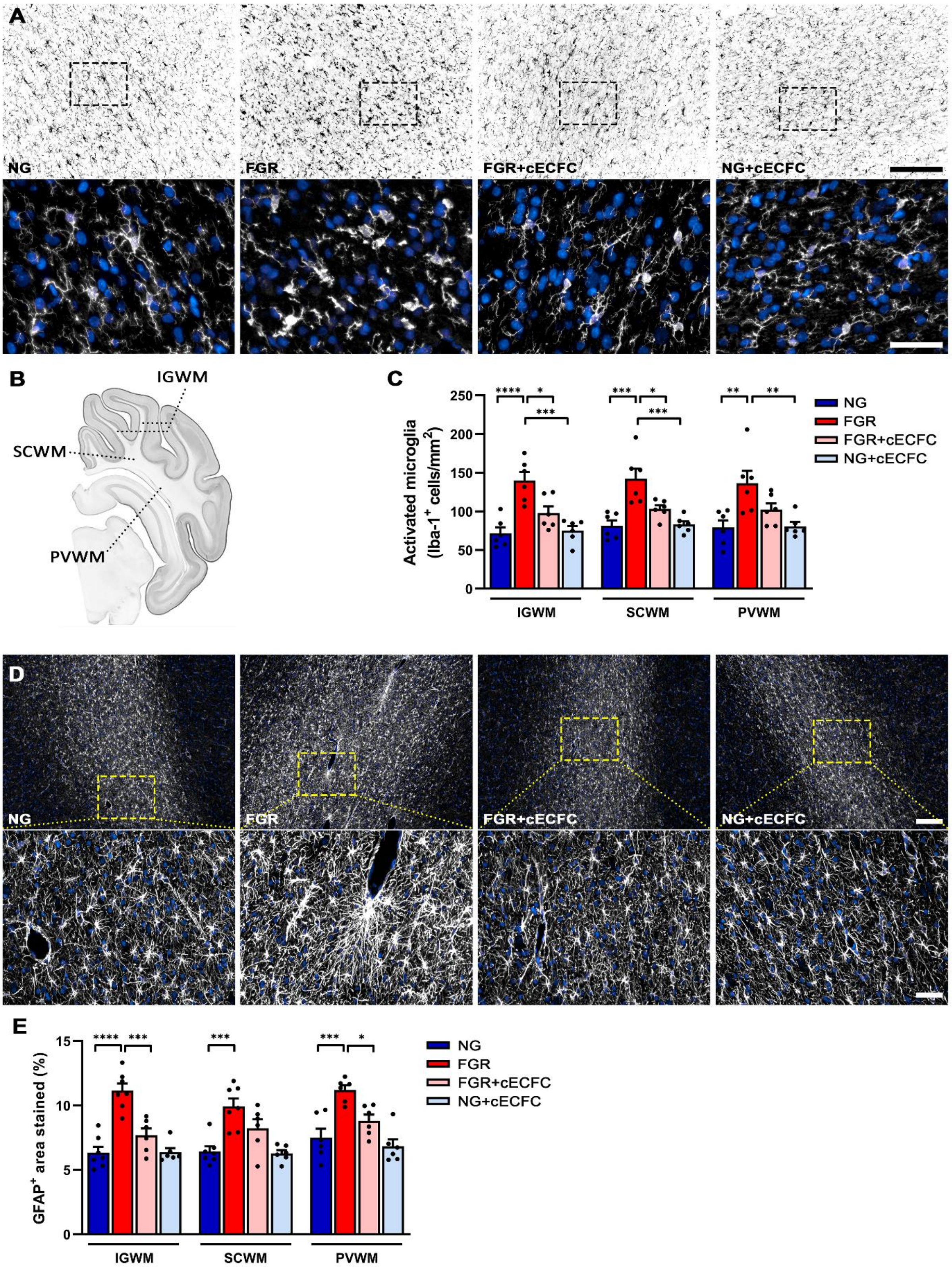

Supplementary Fig. 2. Elevated glial activation in white matter is ameliorated following cECFC treatment in FGR. **(*A*)** Representative labelling of microglia (Iba-1) in the intragyral white matter (IGWM) of NG brain shows even cellular distribution orientated

longitudinally with extended processes, indicative of ramified (resting) microglia. Microglia in FGR white matter demonstrate activated morphology. FGR+cECFC displayed normalised microglia morphology comparable to NG brains. NG+cECFC animals showed no evident alterations in microglia morphology. (***B***) Schematic outline of key white matter regions examined: IGWM, subcortical white matter (SCWM), and periventricular white matter (PVWM). (***C***) FGR brain demonstrated significantly elevated activated microglia counts in all white matter regions when compared with NG brains. Administration of cECFCs significantly ameliorated microglial activation in the IGWM and SCWM but not the PVWM. (***D***) Representative labelling of astrocytes (GFAP) in the intragyral white matter (IGWM) of NG brain shows even cellular distribution of ramified (resting) astrocytes. Astrocytes in FGR white matter demonstrate activated morphology. FGR+cECFC displayed normalised astrocyte morphology comparable to NG brains. Administration of cECFCs to NG animals showed no evident alterations in astrocyte morphology. (***E***) GFAP-positive labelled area was significantly elevated in FGR for all white matter regions when compared with NG brains. Administration of cECFCs significantly ameliorated astrocyte activation in the IGWM and PVWM. All values are expressed as mean +/- SEM (minimum n=6 for all groups). Two-way *ANOVA* with Tukey post-hoc test (*p<0.05, ***p<0.001, ****p<0.0001) (Scale bars: low magnification: 200µm; high magnification: 50µm).

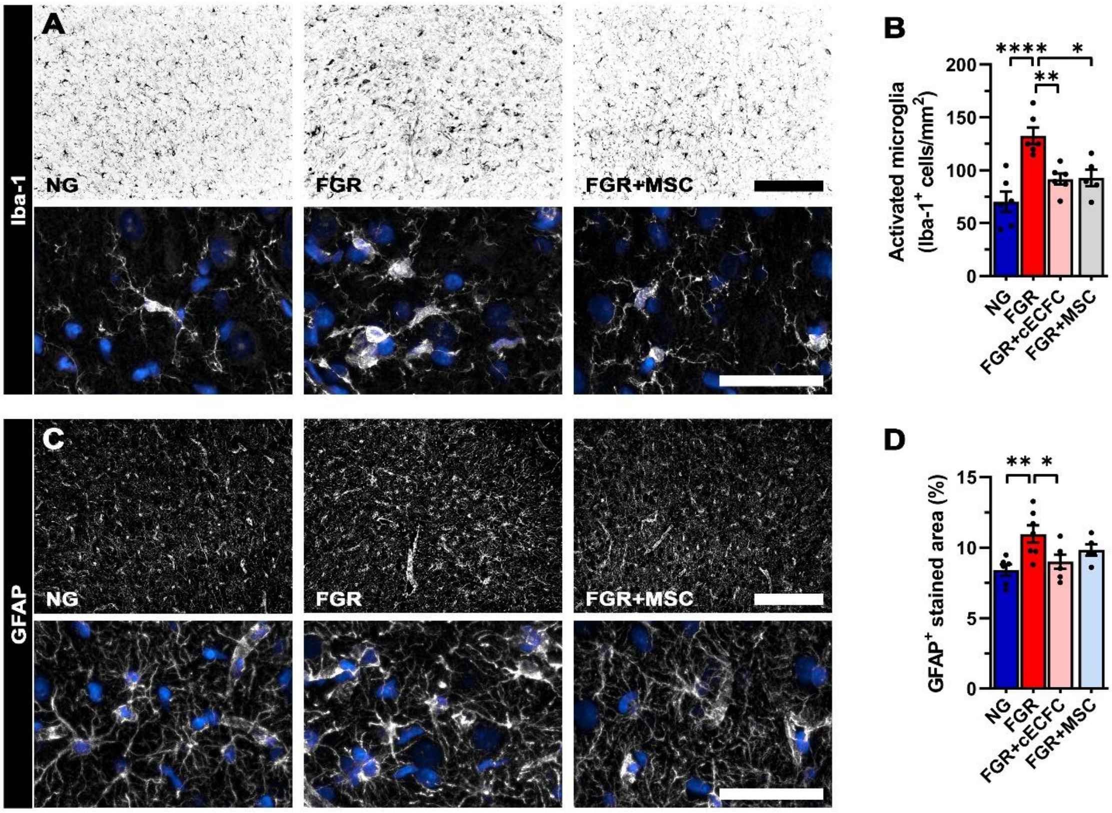

Supplementary Fig. 3. Mesenchymal stromal cell treatment attenuates microglial but not astrocyte activation in FGR brains. ***(A)*** Representative labelling of microglial expression (Iba-1; black) in the parietal cortex. ***(B)*** Treatment with MSCs significantly ameliorated the number of activated microglia. ***(C)*** Astrocytes (GFAP; white) showed evident alterations in morphology. FGR displayed activated astrocyte morphology compared with resting morphology of NG. ***(D)*** MSC treatment did not reduce astrocyte activation as assessed with aerial coverage of GFAP-positive labelled regions compared with untreated FGR. All values are expressed as mean +/- SEM (minimum n=5 for all groups). Two-way *ANOVA* with Tukey post-hoc test (*p<0.05, **p<0.01, ****p<0.0001) (Scale bars: 50 µm; low magnification: 200 µm).

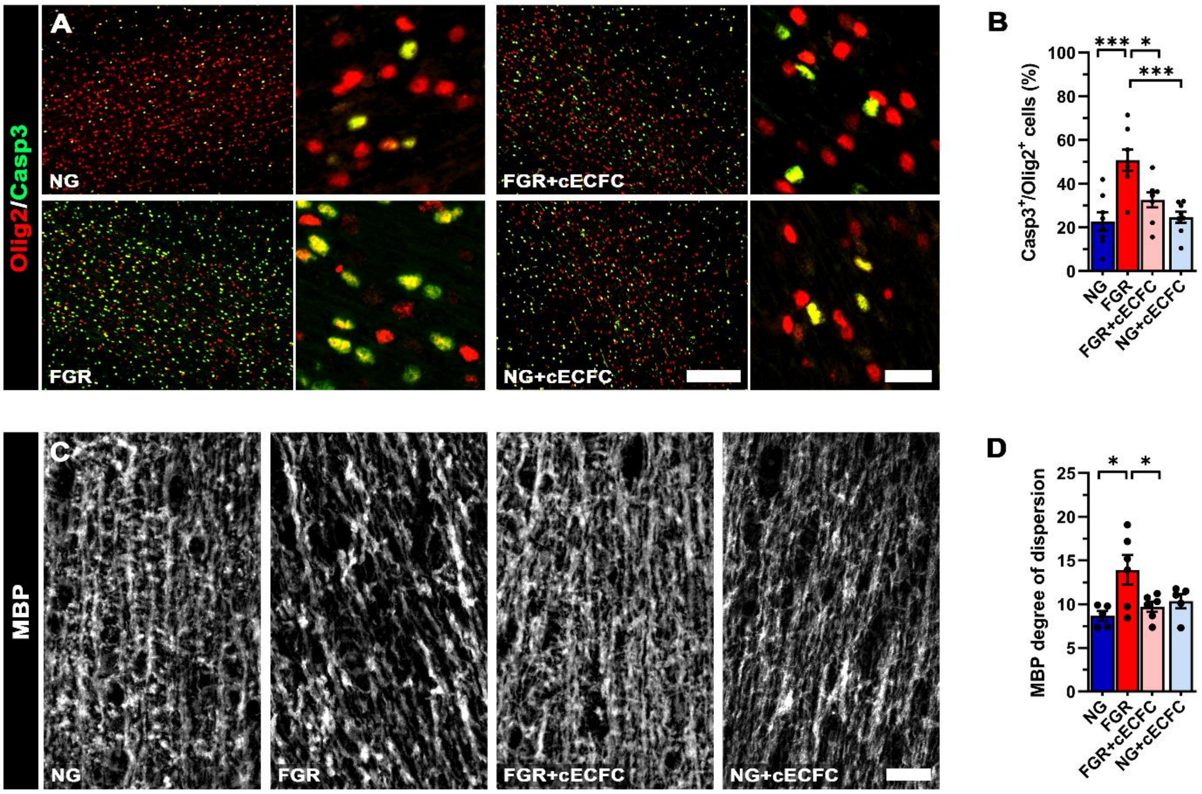

Supplementary Fig. 4. cECFC administration reduces oligodendrocyte apoptosis and improves white matter orientation. (***A***) Increased apoptosis (Casp3; green) of pan oligodendrocytes (Olig2; red) in the IGWM of FGR brain at postnatal day 4. (***B***) Administration of cECFCs significantly decreased apoptosis of oligodendrocytes in FGR compared with untreated FGR. (***C***) Representative labelling of MBP to demonstrate orientation of myelination along axonal fibers in the IGWM. FGR display more diffuse and disorganised labelling patterns compared with all other groups. (***D***) Directionality analysis showed an increase in dispersion of MBP orientation in the IGWM. FGR+cECFC displayed less variable dispersion comparable to that observed in NG and NG+cECFC groups. All values are expressed as mean +/- SEM (minimum *n*=5 for all groups). Two-way *ANOVA* with Tukey post-hoc test (*p<0.05, ***p<0.001) (Scale bars: 50 µm; low magnification A: 200 µm).

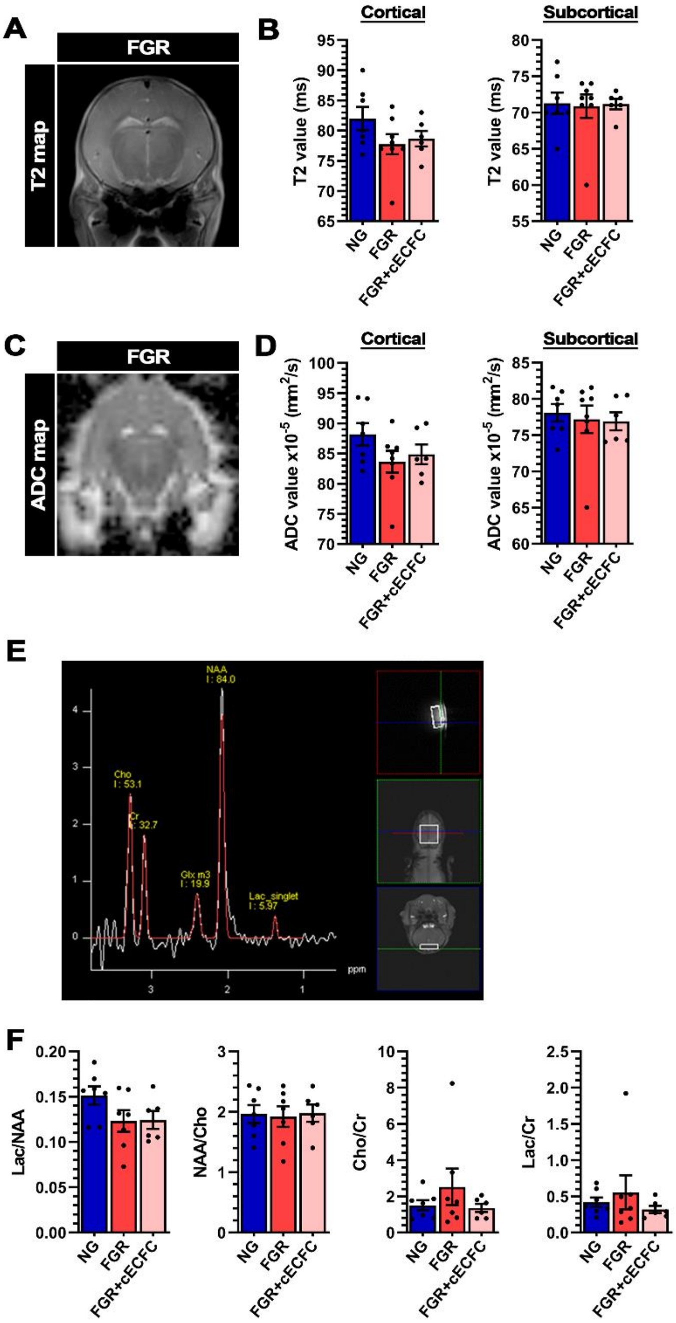

Supplementary Fig. 5. MRI and MRS analyses found no overt differences between NG, FGR and FGR+cECFC animals at day 4. Exemplar coronal views of MRI T2 maps (***A***) and ADC maps **(C)** from an FGR brain at P4. Analysis of T2 and ADC acquisitions showed no significant differences between groups in the cortical or subcortical regions (***B*** & ***D***). (***E***) Representative snapshot of spectrogram showing the fronto-parietal brain voxel used to study brain metabolites. (***F***) No significant differences in metabolite ratios were observed between

NG, FGR, and FGR+cECFC animals. Lactate (Lac), N-Acetylaspartate (NAA), Choline (Chol), Creatine (Cr).

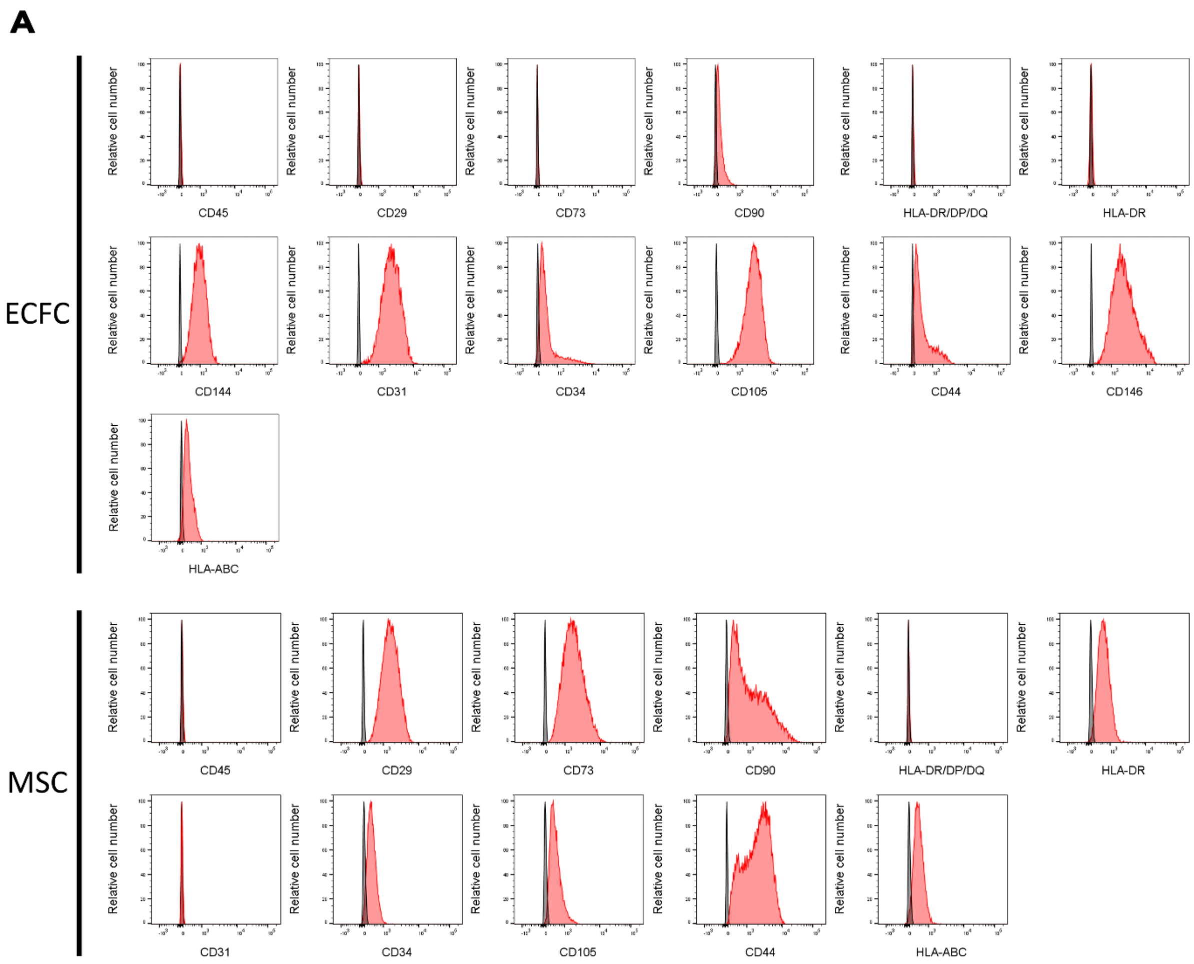

Supplementary Fig. 6. A collection of cell surface markers were used to characterise and confirm ECFC and MSC phenotype via flow cytometry.
