# Supplementary material for: Combination of human endothelial colony-forming cells and mesenchymal stromal cells exert neuroprotective effects in the growth-restricted newborn
Source: NPJ Regen Med. 2021 Nov 18;6:75. doi: 10.1038/s41536-021-00185-5 (PMC8602245; doi:10.1038/s41536-021-00185-5)
Supplement: Supplementary file 2 — Reporting Summary [file 41536_2021_185_MOESM2_ESM.pdf]

nature portfolio

Corresponding author(s): Julie Wixey

Last updated by author(s): Sep 3, 2021

Reporting Summary

Nature Portfolio wishes to improve the reproducibility of the work that we publish. This form provides structure for consistency and transparency in reporting. For further information on Nature Portfolio policies, see our Editorial Policies and the Editorial Policy Checklist.

Statistics

For all statistical analyses, confirm that the following items are present in the figure legend, table legend, main text, or Methods section.

n/a	Confirmed	
☐	☒	The exact sample size (*n*) for each experimental group/condition, given as a discrete number and unit of measurement
☐	☒	A statement on whether measurements were taken from distinct samples or whether the same sample was measured repeatedly
☐	☒	The statistical test(s) used AND whether they are one- or two-sided<br>*Only common tests should be described solely by name; describe more complex techniques in the Methods section.*
☒	☐	A description of all covariates tested
☐	☒	A description of any assumptions or corrections, such as tests of normality and adjustment for multiple comparisons
☐	☒	A full description of the statistical parameters including central tendency (e.g. means) or other basic estimates (e.g. regression coefficient) AND variation (e.g. standard deviation) or associated estimates of uncertainty (e.g. confidence intervals)
☒	☐	For null hypothesis testing, the test statistic (e.g. *F*, *t*, *r*) with confidence intervals, effect sizes, degrees of freedom and *P* value noted<br>*Give P values as exact values whenever suitable.*
☒	☐	For Bayesian analysis, information on the choice of priors and Markov chain Monte Carlo settings
☒	☐	For hierarchical and complex designs, identification of the appropriate level for tests and full reporting of outcomes
☒	☐	Estimates of effect sizes (e.g. Cohen's *d*, Pearson's *r*), indicating how they were calculated

Our web collection on statistics for biologists contains articles on many of the points above.

Software and code

Policy information about availability of computer code

Data collection	Zeiss Zen 2.0 (Blue edition).
Data analysis	FIJI (ImageJ 1.53C; Image Processing and Analysis in Java; National Institutes of Health, Bethesda, MD, USA).<br>Graph Pad Prism 9.0 software (San Diego, California, USA).

For manuscripts utilizing custom algorithms or software that are central to the research but not yet described in published literature, software must be made available to editors and reviewers. We strongly encourage code deposition in a community repository (e.g. GitHub). See the Nature Portfolio guidelines for submitting code & software for further information.

Data

Policy information about availability of data

All manuscripts must include a data availability statement. This statement should provide the following information, where applicable:

- Accession codes, unique identifiers, or web links for publicly available datasets
- A description of any restrictions on data availability
- For clinical datasets or third party data, please ensure that the statement adheres to our policy

The datasets generated during and/or analysed during the current study are available from the corresponding author (JW) on reasonable request.

Field-specific reporting

Please select the one below that is the best fit for your research. If you are not sure, read the appropriate sections before making your selection.

☒ Life sciences ☐ Behavioural & social sciences ☐ Ecological, evolutionary & environmental sciences

For a reference copy of the document with all sections, see nature.com/documents/nr-reporting-summary-flat.pdf

Life sciences study design

All studies must disclose on these points even when the disclosure is negative.

Sample size	Sample size was chosen based on consistent findings and results from our previous studies in the growth restricted piglet brain (Wixey et al., 2019 J Neuroinflamm; Wixey et al., 2019 Front Neurosci)
Data exclusions	No data was excluded from analyses.
Replication	All morphological experiments were successfully replicated (minimum 3 technical replicates for all biological samples).
Randomization	Animals were assigned into FGR or NG groups based on birthweight on day 1. Each pig was then randomly assigned to treatment groups.
Blinding	Administration of stem cells and all analyses were conducted under blinded

Reporting for specific materials, systems and methods

We require information from authors about some types of materials, experimental systems and methods used in many studies. Here, indicate whether each material, system or method listed is relevant to your study. If you are not sure if a list item applies to your research, read the appropriate section before selecting a response.

Materials & experimental systems

n/a	Involved in the study	
☐	☒	Antibodies
☒	☐	Eukaryotic cell lines
☒	☐	Palaeontology and archaeology
☐	☒	Animals and other organisms
☒	☐	Human research participants
☒	☐	Clinical data
☒	☐	Dual use research of concern

Methods

n/a	Involved in the study	
☒	☐	ChIP-seq
☐	☒	Flow cytometry
☐	☒	MRI-based neuroimaging

Antibodies

Antibodies used

Primary Antibody Host Dilution Catalogue#
Anti-human CD34-PE Mouse 1:25 Bio-Rad (MCA1578PE)
Anti-human CD45-FITC Mouse 1:25 BioLegend (304006)
Anti-human CD31-V450 Mouse 1:30 BD Biosciences (561653)
Anti-human CD105-PE Mouse 1:50 BD Biosciences (560839)
Anti-human CD144-FITC Mouse 1:50 BD Biosciences (560411)
Anti-Human CD90-PE.CY5 Mouse 1:800 BD Biosciences (555597)
Anti-Human CD44-FITC Mouse 1:50 BD Biosciences (347943)
Anti-Human CD29-BV480 Mouse 1:50 BD Biosciences (746592)
Anti-Human CD73-FITC Mouse 1:50 BD Biosciences (561254)
Anti-Human CD146-FITC Mouse 1:50 BD Biosciences (560846)
Anti-Human HLA-ABC-PE Mouse 1:50 BD Biosciences (565291)
Anti-Human HLA-DR-PE Mouse 1:50 BD Biosciences (567054)
Anti-Human HLA-DR/DP/DQ-FITC Mouse 1:50 BD Biosciences (555558)
7AAD 1:40 BD Pharmingen (559925)
Lamin A/C Rabbit 1:200 Abcam (ab108595)
Endothelial progenitor cells (CD34) Goat 1:1000 R&D Systems (AF3890)
Collagen IV (Col IV) Rabbit 1:1000 Abcam (ab6586)
Endothelial cells (CD31) Rabbit 1:500 Abcam (ab28364)
Albumin Sheep 1:1000 Abcam (ab8940)
IgG Goat 1:1000 JIR (114-005-003)
S100B Rabbit 1:1000 Invitrogen (PA5-78161)
Ionised calcium binding adaptor molecule-1 (Iba-1) Goat 1:1000 Abcam (ab5076)
Glial fibrillary acidic protein (GFAP) Mouse 1:1000 Sigma (G3893)

	Glial fibrillary acidic protein (GFAP) Rabbit 1:2000 DAKO (Z0334)<br>Neuronal Nuclei (NeuN) Rabbit 1:1000 Abcam (ab177487)<br>Microtubule-associated protein 2 (MAP2) Mouse 1:500 Sigma-Aldrich (M4403)<br>Cleaved Caspase-3 Rabbit 1:500 Cell Signaling (#9661)<br>Caspase-9 Rabbit 1:500 Abcam (ab32539)<br>Myelin Binding Protein (MBP) Rat 1:1000 Abcam (ab7349)<br>Neurofilament (NF) Mouse 1:500 Abcam (ab134306)<br>Oligodendrocyte marker 2 (Olig2) Rabbit 1:1000 Genetex (GTX132732)
Validation	Antibodies used in FACS are human specific as per manufacturer's website.<br>Immunohistochemistry studies included negative controls (omission of primary antibody). Where pig specificity was not validated by manufacturer's, we utilised antibodies with reactivity to human or bovine due to close homology to porcine. Validation was also conducted using positive controls (tissue known to express the appropriate marker). Antibodies have also been used in previous publications (Wixey et al. 2019a&b, Goasdoue et al. 2019).

Animals and other organisms

Policy information about studies involving animals; ARRIVE guidelines recommended for reporting animal research

Laboratory animals	Large White piglets (n=36), equal mixed sex. Litter matched from multiple sows (n=14).
Wild animals	Study did not involve wild animals.
Field-collected samples	Study did not involve samples collected from the field.
Ethics oversight	Approval for this study was granted by The University of Queensland Animal Ethics Committee (UQCCR/420/17) and was carried out with respect to the National Health and Medical Research Council guidelines (Australia) and ARRIVE guidelines. Animals care was in accordance with institutional guidelines.

Note that full information on the approval of the study protocol must also be provided in the manuscript.

Flow Cytometry

Plots

Confirm that:

☒ The axis labels state the marker and fluorochrome used (e.g. CD4-FITC).

☒ The axis scales are clearly visible. Include numbers along axes only for bottom left plot of group (a 'group' is an analysis of identical markers).

☒ All plots are contour plots with outliers or pseudocolor plots.

☒ A numerical value for number of cells or percentage (with statistics) is provided.

Methodology

Sample preparation	The cells were cultured in an invitro setting, before being passaged and suspended in flow cytometry medium ready for acquisition through the machine
Instrument	FACSAria Fusion (Becton Dickinson)
Software	FloJo
Cell population abundance	There is just 1 population to assess at a time when determining cell surface marker expression
Gating strategy	Negative and positive controls were used to assess cell surface marker expression as well as the position gating strategy to identify positive and negative populations

☒ Tick this box to confirm that a figure exemplifying the gating strategy is provided in the Supplementary Information.

Magnetic resonance imaging

Experimental design

Design type	Resting state.
Design specifications	Single imaging session undertaken on postnatal day 4.
Behavioral performance measures	No tasks were undertaken as animal was sedated. MRI was conducted solely for structural study.

Acquisition

Imaging type(s)	Structural
Field strength	7T
Sequence & imaging parameters	150mm volumetric head coil was used to acquire sagittal, axial and coronal high-resolution slices. The brain proton signal was used to perform shimming and improve field homogeneity. The sequence parameters were: TR/TE 8500/60 ms, 1.6 mm slice thickness, acquisition matrix 64x64, with 4 b values (0, 700, 1500 and 3000 s/mm2, with 3, 20, 30 and 64 gradient directions respectively). To assist in the correction for image distortions induced by magnetic susceptibility inhomogeneities, 3 additional non-diffusion weighted images were acquired along the reverse phase encoding direction (PA).
Area of acquisition	Regions of interest were defined manually

Diffusion MRI ☐ Used ☒ Not used

Preprocessing

Preprocessing software	3D Slicer 4.10.2 (Linear transform), FSL
Normalization	Linear transform in 3 dimensions was used to align each brain map.
Normalization template	No template was used.
Noise and artifact removal	The b=0 weighted image in the anterior-posterior (AP) and reverse (PA) direction were combined to calculate an image distortion map that was subsequently used to correct for susceptibility-induced distortions. Individual diffusion weighted images were then corrected for head motion between DWI volumes and geometric distortions due to magnetic susceptibility inhomogeneities and eddy currents using FSL Eddy.
Volume censoring	No volume censoring was used.

Statistical modeling & inference

Model type and settings	Ordinary one-way ANOVA with Tukey post-hoc test was used.
Effect(s) tested	Group effect was tested

Specify type of analysis: ☐ Whole brain ☒ ROI-based ☐ Both

Anatomical location(s)	Anatomical locations were manually determined
Statistic type for inference (See Eklund et al. 2016)	Voxel-wise.
Correction	No correction was applied.

Models & analysis

n/a	Involved in the study
☒	☐ Functional and/or effective connectivity
☒	☐ Graph analysis
☒	☐ Multivariate modeling or predictive analysis
